# Supplementary material for: Current Treatment Methods for Charcot–Marie–Tooth Diseases
Source: Biomolecules. 2024 Sep 9;14(9):1138. doi: 10.3390/biom14091138 (PMC11430469; doi:10.3390/biom14091138)
Supplement: Supplementary file 1 [file biomolecules-14-01138-s001.zip › biomolecules-3141920-supplementary.pdf]

Table S1. Cellular Mechanisms and Associated Presentation of CMT1

Type (OMIM)	Gene	Presumed disease mechanism	Presentation
CMT1A (118220), AD	*PMP22*	Impaired regulation of myelin biosynthesis, alteration of mRNA processing, demyelination[1]	Foot deformity (High arches and hammer toes), Sensory deficits[2]
CMT1B (118200), AD	*MPZ*	Impaired myelin compaction, retention in endoplasmic reticulum (ER) and unfolded protein response[3]	Indistinguish with CMT1A[4], Bilateral foot drop, Scoliosis, Ambulatory disturbance[5-7]
CMT1C (601098), AD	*LITAF*	Impaired protein degradation in early endosomes[8]	Foot deformity, mild gait dysfunction, leg cramping or pain, and mild sensory symptoms[9]
CMT1D (607678), AD	*EGR2*	Impaired expression of myelin-related genes[10]	Pes cavus, distal lower limbs weakness, Sensory symptoms, hand weakness and atrophy, scoliosis[10]
CMT1E (118300), AD	*PMP22*	Retention and accumulation of mutant PMP22 in ER, unfolded protein response[11]	Pes cavus, weakness of the tibialis anterior muscles, sensory deficits (No significant disabilities)[12-14]
CMT1F (607734), AD	*NEFL*	Aberrant neurofilament assembly and transport, protein aggregations[15]	Pes cavus, steppage gait, hammer toes but was able to walk without assistance[16]
CMT1G (618279), AD	*PMP2*	Aberrant transport of fatty acids, impaired organization of compact myelin, short internodes[17]	Pes cavus without scoliosis, disability of the upper and lower limbs, Areflexia[17]

Table S2. Cellular Mechanisms and Associated Presentation of CMT2

Type (OMIM)	Gene	Presumed disease mechanism	Presentation
CMT2A1 (118210), AD	*KIF1B*	Impaired transportation of IGF1R to the axon[18]	Bilateral pes cavus, hammertoes, and mild lordosis, moderate muscle weakness and atrophy of the anterior tibial, peroneal, and posterior tibial muscles[18,19]
CMT2A2 (609260), AD	*MFN2*	Disturbance of mitochondrial fusion[20]	Foot deformity, limping gait, pyramidal signs, distal muscle weakness and wasting, mild distal sensory loss[19,21,22]
CMT2B (600882), AD	*RAB7*	Impaired endosomes trafficking and axonal integrity[23]	Foot painful ulcer with swelling and deformity, distal weakness and wasting, moderate distal sensory loss[24,25]
CMT2B1 (605588), AR	*LMNA*	Lamin A/C nuclear-envelope proteins synthesis defect and abnormal axon structure[26,27]	Pes cavus, distal muscle weakness and wasting, lower limb areflexia[28]
CMT2B2 (605589), AR	*MED25/PNKP*	Increased DNA damage with subsequent cell death[29]	Weakness and atrophy in the ankles, hyporeflexia, mild ataxia and sensory deficits in a symmetrical 'stocking-glove' pattern[29-31]

CMT2C (606071), AD	*TRPV4*	Cation channel mediates calcium influx results in peripheral axonal degeneration[32,33]	Muscle weakness of limbs[34], vocal cords, intercostal muscles, pes cavus[35], foot drop[36]
CMT2CC (616924), AD	*NEFH*	The addition of cryptic amyloidogenic elements (CAE) results in the disruption of neurofilament network[37]	Distal muscle weakness and atrophy, high-arched feet, hyporeflexia, and distal sensory impairment[37]
CMT2D (601472), AD	*GARS1*	Glycyl tRNA synthetase defect impaired the protein translation[38,39]	Distal muscle weakness and atrophy with sensory loss[40], more severe in the hands[41], steppage gait[42], pes cavus and hammertoes[41]
CMT2DD (618036), AD	*ATP1A1*	Reduction in Na+/K (+)-ATPase activity causing increased intracellular axonal Ca (2+) levels result in axonal degeneration[43]	Distal muscle weakness and atrophy of lower limbs, decreased vibratory sensation, foot drop, pes cavus[43]
CMT2E (607684), AD	*NEFL*	Disruption of the neurofilament network, motor neuron degeneration[44]	Pes cavus, plantar extensor weakness[45], distal lower limb atrophy and weakness, hammertoes[46], ataxic gait[47]
CMT2EE (618400), AR	*MPV17*	Disturbance of mitochondrial DNA maintenance[48]	Severe distal muscle atrophy, ankle joint instability, pes cavus, claw toes, decreased muscle strength in the hands[49]
CMT2F (606595), AD	*HSPB1*	Disruption of the neurofilament network, motor neuron degeneration[44]	Foot drop, steppage gait, clawing of the hands, talipes cavus, mild to moderate sensory impairments[50,51]
CMT2FF (619519), AD	*CADM3*	Disturbance of the axon-glia interface[52]	Foot drop, weakness and atrophy of lower limbs, scoliosis, facial weakness[52]
CMT2GG (606483), AD	*GBF1*	Disfunction of the Golgi apparatus and intracellular vesicular trafficking or cargo movement[53]	Foot drop, steppage gait, distal sensory impairment, hammertoes and pes cavus[53]
CMT2H (607731), AR	*GDAP1*	Decreased mitochondrial dynamic[54,55]	Pyramidal features, severe muscle wasting[56]
CMT2HH (619574), AD	*JAG1*	Disruption of peripheral nerve integrity[57]	Distal limb weakness， tremor, distal sensory loss and areflexia, pes cavus, scoliosis , kyphoscoliosis, vocal cord paresis[57]
CMT2I (607677), AD	*MPZ*	Impaired myelin compaction, retention in endoplasmic reticulum (ER) and unfolded protein response[58]	Distal muscle weakness and atrophy, foot deformity, distal sensory loss, hyporeflexia gait abnormalities[59]
CMT2II (620068), AD	*SLC12A6*	Impaired cell volume homeostasis in peripheral nerves, secondary axonal degeneration or loss, altered neuronal excitability[60]	Delayed walking, frequent falls, foot dragging, foot drop[61], and distal muscle weakness and mild atrophy[62]

CMT2J (607736), AD	*MPZ*	Impaired myelin compaction, retention in endoplasmic reticulum (ER) and unfolded protein response[58]	Marked sensory abnormalities, deafness, and pupillary abnormalities[63], hypertrophy of calf muscles with absent Achilles tendon reflexes[64]
CMT2K (607831), AD, AR	*GDAP1*	Decreased mitochondrial dynamic[54,55]	Difficulty walking, foot deformities, kyphoscoliosis, distal limb muscle weakness and atrophy, areflexia, and diminished sensation in the lower limbs[65,66]
CMT2L (608673), AD	*HSPB8*	Protein misfolding[67,68]	Bilateral pes cavus, hammertoes, and mild lordosis, moderate muscle weakness and atrophy of the anterior tibial, peroneal, and posterior tibial muscles[18,19]
CMT2M (606482), AD	*DNM2*	Endosomal trafficking[69]	Pes cavus, clawed toes, wasting of the peroneal muscles, steppage gait with sensory ataxia[70]
CMT2N (613287), AD	*AARS1*	Glycyl tRNA synthetase defect impaired the protein translation[38,39]	Distal muscle weakness and atrophy of the lower limbs, hyporeflexia of the distal lower limbs, and foot deformities[71]
CMT2O (614228), AD	*DYNC1H1*	Impaired axonal transport[72]	Distal lower limb weakness and wasting with pes cavus deformity[73]
CMT2P (614436), AD, AR	*LRSAM1*	Disturbed neurodevelopment, less organized neural structure[74]	Pes cavus, toe clawing, stocking hypoesthesia, ankle areflexia, and amyotrophy[74,75]
CMT2Q (615025), AD	*DHTKD1*	Decreased ATP synthesis[76]	Deep sensory impairment, wasting and weakness of the lower limbs, decreased or absent deep tendon reflex[76]
CMT2R (615490), AR	*TRIM2*	Impaired axonal transport[77]	Atrophy of the small muscles in the hands and feet, and areflexia broad-based gait, pes cavus, vocal cord paralysis[78,79]
CMT2S (616155), AR	*IGHMBP2*	Impaired the capacity of neurons to produce error-free mature mRNA, leading to neuronal degeneration[80]	Toe walking, foot drop, and steppage gait[80], pes equinovarus, scoliosis and mild proximal muscle weakness, abnormal tongue shape, absent reflexes and distal sensory impairment[81]
CMT2T (617017), AD, AR	*MME*	Neuronal degeneration[82]	Weakness and atrophy of the distal lower limb muscles, distal sensory impairment and hyporeflexia[83]
CMT2U (616280), AD	*MARS1*	Impaired axonal transport[84]	Steppage gait, distal muscle wasting and weakness in the upper and lower limbs[84], distal sensory impairment, absence of distal deep tendon reflexes[85]

CMT2V (616491), AD	*NAGLU*	Unknown	Painful sensory neuropathy, distal upper limb paresthesia, decreased vibration sense and loss of deep tendon reflexes[86]
CMT2W (616625), AD	*HARS1*	Axonal degeneration[87]	Distal lower limb weakness and atrophy, pes cavus, kyphoscoliosis, ankle contractures[88]
CMT2X (616668), AR	*SPG11*	Impaired cargo trafficking[88]	Distal lower limb and intrinsic hand muscles weakness and atrophy, pes cavus, kyphoscoliosis, ankle contractures, tremor[88]
CMT2Y (616687), AD	*VCP*	Impaired autophagic degradation of mitochondria or mitophagy[89,90]	Proximal and distal lower limb weakness, proximal upper limb weakness, pes cavus, hammertoes, tight heel cords, lordosis, scapular winging, toe-walking, distal sensory impairment wide-based gait, dysarthria, dyspnea, mood and behavioral abnormalities[89,90]
CMT2Z (616688), AD	*MORC2*	Impaired DNA repair and gene regulation[91]	Distal weakness and wasting of the lower limbs with sensory loss[92], pes cavus, hammertoes, pyramidal signs[91], spastic dysphonia, hearing loss, neck weakness, claw hands[93]

Table S3. Cellular Mechanisms and Associated Presentation of CMT4

Type (OMIM)	Gene	Presumed disease mechanism	Presentation
CMT4A (214400), AR	*GDAP1*	Increased the production of ROS[94]	Distal weakness and atrophy of the limbs, Sensory loss with abolished deep tendon reflexes[95]
CMT4B1 (601382), AR	*MTMR2*	Disturbed modulation of membrane trafficking[96]	Similar with CMT4B2 phenotype
CMT4B2 (604563), AR	*SBF2*	Impaired the folding of the myelin sheaths[97]	Distal lower limb weakness and atrophy[98], distal sensory loss, areflexia, bilateral pes equinovarus, hearing loss[97]
CMT4B3 (615284), AR	*SBF1*	Impaired membrane trafficking and endosome function[99]	Distal lower extremity weakness and atrophy[100], pes planus, areflexia, distal sensory loss, scoliosis, microcephaly and cognitive impairment[101]
CMT4C (601596), AR	*SH3TC2*	Impaired intracellular vesicular trafficking[102]	distal muscle atrophy and weakness, areflexia, foot deformities (pes cavus and hammer toes, clubfoot deformity), and distal sensory impairment[103], severe

			scoliosis[104] ,kyphoscoliosis with deafness, slow pupillary light reflexes, and lingual fasciculations[105]
CMT4D (601455), AR	*NDRG1*	Impaired endosomal transportation and inefficient trafficking [106,107]	Distal muscle wasting and atrophy, talipes cavus equinovarus, pes cavus, hammertoes, and claw hands, tendon areflexia, and sensory loss[108], kyphoscoliosis, deafness[109,110]
CMT4E (605253), AR	*EGR2/MPZ*	Impaired expression of myelin-related genes[10]	Distal muscle wasting, steppage gait, foot drop[111]
CMT4F (614895), AR	*PRX*	Impaired maintenance of peripheral nerve myelin[112-114]	Distal sensory impairment and distal muscle weakness and atrophy[115] affecting the lower more than the upper limbs[116], pes cavus, scoliosis, absent reflexes, sensory ataxia[117]
CMT4H (609311), AR	*FGD4*	Impaired Rho GTPase signaling, incorrect myelination of axons[118]	Distal muscle weakness and atrophy, steppage gait, pes equinovarus, pes cavus[119], lower limb areflexia, and distal sensory loss, scoliosis, pupil size asymmetry[120]
CMT4J (611228), AR	*FIG4*	Impaired autophagy in astrocytes and neurons[121,122]	Progressive distal and proximal muscle weakness with areflexia[121]<br>cranial nerve dysfunction, such as eye abduction or tongue movement abnormalities[123,124]
CMT4K (616684), AR	*SURF1*	Impaired COX assembly and reduced COX activity[125-127]	Easy fatigability, kyphoscoliosis, muscle atrophy of the hands and feet, and distal sensory, impairment of the lower limbs[127]

Table S4. Cellular Mechanisms and Associated Presentation of CMTX

Type (OMIM)	Gene	Presumed disease mechanism	Presentation
CMTX1 (302800), XLD	*GDAP1*	Increased the production of ROS[94]	Distal limb atrophy and weakness, intrinsic hand muscles atrophy, pes cavus, scoliosis[128], distal sensory abnormalities, toe walking, Achilles contractures, hand tremor, depressed deep tendon reflexes, gait and limb ataxia, hearing loss, CNS symptoms included paresis, dysarthria, aphasia, and cranial nerve palsies[129,130]
CMTX2 (302801), XLR	*Unknown*	Unknown	Atrophy and weakness of lower leg muscles, areflexia, and pes cavus[131]

CMTX3 (302802), XLR	*Unknown*	Unknown	Distal muscle atrophy and weakness and distal sensory loss[132], equinovarus foot deformity, pes cavus and areflexia[133], scoliosis, pain<br>spastic paraparesis[134]
CMTX4 (310490), XLR	*AIFM1*	Abnormal mitochondrial morphology and accumulation[135-137]	Distal weakness, muscle atrophy, sensory loss, areflexia, pes cavus, and hammertoes, deafness and cognitive impairment,<br>hearing loss[138], optic nerve atrophy and retinopathy with further visual loss[139]<br>cerebellar dysfunction, including dysarthria and intention tremor[140].
CMTX5 (311070), XLR	*PRPS1*	Impaired the synthesis of PRPP and ATP production[141]	Distal weakness and atrophy in all extremities, pes cavus, with broad-based gait[142], atrophy of the intrinsic hand muscles[143], absent deep tendon reflexes, distal sensory impairment[144].<br>hearing loss, retinitis pigmentosa, progressive loss of vision beginning with optic atrophy[145]
CMTX6 (300905), XLD	*PDK3*	Impaired ATP production and lactate accumulation[146]	Distal lower limb muscle weakness and atrophy, sensory abnormalities, hand muscle weakness, hand tremor, decreased ankle reflexes, pes cavus, hearing loss[146]

Table S5. Details of exercise prescription for CMT patients

Intervention	Load, repetitions and sets	Frequency and duration	Ref
Strengthening exercises			
Upper limbs			
1) Elbow flexion<br>2)Elbow extension<br>3) Hand grip	1) 20-30% MVIC, 3 sets, 4-10 reps<br>2) 20-30% MVIC, 3 sets, 4-10 reps<br>3) 100% MVIC, 3 sets, 4 reps<br>1-min rest between sets for all	3 times per week<br>12 weeks	[147,148]
Lower limbs			
1) Hip extension<br>2) Hip abduction	For 1) to 4)<br>60% 1RM, 3sets,25 reps (1-8w)	For 1) to 4)<br>3 times per week	[150-154]

3) Knee extension<br>4) Knee flexion<br>5) Hip flexion[149]<br>6) Ankle dorsiflexion	70% 1RM, 3 sets,15 reps (9-16w)<br>80% 1RM, 3 sets, 10 reps (18-24w)<br>For 5)<br>40-60% MVIC, 2 sets, 8-12 reps<br>For 6)<br>60-80% or 50-70% 1RM, 2-3 sets, 10 reps<br>1-min rest between sets for all	24 weeks<br>For 5)<br>4 times per week<br>16 weeks<br>For 6)<br>3 times per week<br>12 weeks	
Aerobic exercises			
Cycling	1) 70% of VO_2 max, 6 x 5 min bouts 2 min rest between each bout<br>2) 5min warm up, 6 x 5 min bouts (4 min at 40% Pmax and 1 min at 80% Pmax), 10min cool down, progressive increase in exercise intensity at 80% Pmax to maintain heart rates within 70-90% MHR	For 1)<br>3 times per week<br>12 weeks<br>45 min<br>For 2)<br>3 times per week<br>24 weeks<br>45 min	[155]<br>[156]
Walking	15 to 30min walk to maintain heart rates within 50- 60% MHR	3 to 4 times per week<br>12 weeks	[157]
Combination exercises			
1) Treadmill,<br>2) Respiratory<br>3) Proprioceptive exercises	1) Treadmill (30 min) – walking from 40 to 70% maximal load<br>2) Respiratory rehabilitation (25 min)-PEP-and the expiration with ELTGOL<br>3) Proprioceptive exercise (25 min) - Perfetti method.	2 times per week<br>8 weeks<br>90 min	[158,159]
1) Passive stretching<br>2) Muscle strengthening<br>3) Dynamic balance training	1) Passive stretching (10 min)<br>2) Muscle strengthening (10 min)<br>3) Balance training during standing and stepping (20 min)	6 times per week<br>2 weeks<br>40 min	[160]

Table S6. The therapeutic strategies for CMT neuropathies

Type	Compound	Mechanism	Test model	Outcome	Ref
Drug Therapies					
CMT1A	PXT3003	Decreased PMP22 expression	CMT1A Rat (Oral)	Hindlimb muscle strength increase	[161,162]

	Onapristone	Decreased PMP22 expression	CMT1A Rat (Intraperitoneal)	Improved motor performance	[163,164]
	Ascorbic acid	Decreased PMP22 expression	C22 Mouse (Oral)	Improved locomotion	[165,166]
	Fasting diet	Improved autophagy	Trembler J (Tr-J) Mouse	Maintain the locomotor performance	[167]
	AUY922	Hsp90 inhibition	Tr-J Mouse (Intraperitoneal)	Prevented neuromuscular degeneration	[168]
	A438079	P_2X_7 inhibitor to reduce abnormal Ca^{2+} influx into SC	CMT1A Rat (Intraperitoneal)	Improved muscle strength	[169,170]
	rhNRG1	PI3K/AKT pathway activation	CMT1A Rat (Intraperitoneal)	Improved motor performance	[171]
	Lipids	Promoted lipid metabolism	CMT1A Rat (Oral)	Improved motor performance	[172]
	Curcumin	ER-stress reduction and decreased UPR activation	CMT1A Rat (Intraperitoneal)	Improved sensori-motor functions and nerve conduction	[173]
	Sephin1	Inhibition of eIF2A dephosphorylation by GADD34	C3-*PMP22* Mouse (Oral)	Improved motor capacity and MNCV	[174]
	PLX5622	Decreased CSF1R inhibitor	C3-*PMP22* Mouse (Intraperitoneal)	Ameliorated muscle strength	[175]
	CKD-504	HDAC6 inhibition: decreased Hsp90 acetylation	C22 Mouse (Oral)	Improved motor function and sensory-motor balance	[176]
	RGFP966	HDAC3 inhibition: activated myelin protein expression	C3-*PMP22* Mouse (Sub-cutaneous and intraperitoneal)	Improved the motor performance	[177]
CMT1B	Curcumin	ER-stress reduction and decreased UPR activation	R98C Mouse (Oral)	Evaluated strength of all limbs	[178]
	Sephin1	Inhibition of eIF2A dephosphorylation by GADD34	R98C Mouse (Oral)	Improved motor capacity and MNCV	[174,179]
	Sildenafil	Increased proteasome activity	*S63*del Mouse (Intraperitoneal)	Improved motor performance	[180]
	PLX5622	Decreased CSF1R inhibitor	*P0*het Mouse (Intraperitoneal)	Preserved motor function	[181]
	NRG1-III	Activated myelin genes	S63del Mouse (Intraperitoneal)	Improved motor function	[182]
CMT1E	Fasting diet	Improved autophagy	Tr-J Mouse	Maintain the locomotor performance	[167]
	Rapamycin	Improved autophagy	Tr-J Mouse (Oral or Intraperitoneal)	No locomotor performance improved	[183]
	Curcumin	ER-stress reduction and decreased UPR activation	Tr-J Mouse (Oral)	Improved motor performance	[184,185]

CMT2A	MiM111	Increased function of MFN2	*Mfn2*^{T105M} Mouse (Oral)	Maintain the motor performance	[186]
	MFN1	Increased MFN1 activity	*Mfn2*^{R94Q} Cells	-	[187]
	SARM1 KO	Decreased SARM1 NADase activity	*Mfn2*$^{H361Y/+}$ Rats	Prevented muscle atrophy	[188]
	MFN2 agonists	Improved mitochondrial trafficking	*Mfn2*^{T105M} Mouse	-	[189]
CMT2D	Tubastatin-A	HADC6 inhibitor: increased tubulin acetylation	*Gars*$^{P234KY/+ \text{ or } C201R/+}$ Mouse	Improved motor functions	[190,191]
CMT2E	Serine/threonine Kinase inhibitors	Partially reverse neurofilament deposits phenotype	CMT2E-iPSCs	-	[192]
CMT2F	Tubastatin-A	HADC6 inhibitor: increased tubulin acetylation	CMT2F-iPSCs	-	[191,193]
	ACY-738,775,1215	HADC6 inhibitor: increased tubulin acetylation	*HSPB1*^{S135F} Mouse (Intraperitoneal)	Reversed the motor and sensory	[194]
CMT4B1	Rapamycin	mTORC1 inhibitor: increased myelin production	*Rab35*$^{flox/+}$ Mouse (Intraperitoneal)	Rescued impaired myelin segment formation and ameliorated aberrant myelin	[195]
	Niaspan	Downregulated Nrg1 type III signaling	*Mtmr2*$^{-/-}$ Mouse (Intraperitoneal)	Reduced myelin outfolding	[196]
	PIKfyve inhibitors	To decrease PI3,5P2 levels	*Mtmr2*$^{-/-}$ KO Mouse	Reduced myelin outfolding	[197]
CMTX1	PLX5622	Decreased CSF1R inhibitor	*Cx32*def Mouse (Intraperitoneal)	Ameliorated muscle strength	[175]
Gene Therapies					
CMT1A	AAV1/*NT-3*	Neurotrophic activity	Tr-J Mouse (Muscular Injection)	Improvement motor function	[198,199]
	AAV2/9 shRNA	Decreased PMP22 expression	C57BL/6 Mouse (Intraneural) CMT1A Rats (Intraneural)	Prevented motor and sensory defects	[200]
	scAAV9 miRNA	Decreased PMP22 expression	C61het Mouse (Intraneural)	Improved the motor performance	[201]
	ASOs	Decreased PMP22 expression	C22 Mouse (Subcutaneous)	Reversed motor deficits	[202]
	siRNA SQ NPs	Decreased PMP22 expression	JP18/JY13 Mouse (Intravenous)	Restored locomotor activity	[203]
	siRNA	To target mutated allele	Tr-J Mouse (Intraperitoneal)	Improved anatomical, physiological, and behavioral	[204]

	CRISPR/Cas9 RNP	Decreased PMP22 expression	C22 Mouse (Intraneural)	Increased MNCV	[205]
	Lentiviral/miR-318	Decreased PMP22 expression	C22 Mouse (Intraneural)	Prevented the behavioral deficits	[206]
	AAV2/miR-29a	Decreased PMP22 expression	C22 Mouse Schwann Cells	-	[207]
	ATFO	Decreased PMP22 expression	Schwann cell	-	[208]
CMT1E	siRNA	To target mutated allele	Tr-J Mouse (Intraperitoneal)	Improved anatomical, physiological, and behavioral	[204]
CMT2A	AAV8/*SARM1* mutants	Block the wild type *SARM1* function	*SARM1*-KO Mouse (Intraneural)	Block pathological axon degeneration	[209]
	*MFN1*genetic addition	Compensate mutated *MFN2* dysfunction	*MFN2*^{R94Q} Mouse	Restored sensorimotor function	[210]
CMT2D	scAAV9 RNAi	To target allele specific mutations in *GARS*	*Gars*$^{\Delta ETAQ\ or\ huEx8}$ Mouse	Prevented deficits in gross motor performance	[211]
	AAV1/*NT-3*	Neurotrophic activity	*Gars*$^{P278KY/+}$Mouse (Intramuscular)	Improved in the rotarod test and toe spreading	[212]
CMT2S	AAV9/ *IGHMBP2*	Restored *IGHMBP2* gene function	AAV9-*IGHMBP2- nmd* Mouse	Improved neuromuscular function and survival	[213,214]
CMT4C	Lentiviral/ *SH3TC2*	Restored *SH3TC2* gene expression	*Sh3tc2*$^{-/-}$ Mouse (Intraneural)	Improved motor performance and nerve function	[215]
CMT4J	AAV9/ *FIG4*	Restored *FIG4* expression	*Fig4*$^{plt/plt}$ Mouse (Injection Cerebral Ventricle)	Prolonged life span and improved peripheral nerve function	[216]
CMTX1	Lentiviral/*GJB1*	Cx32 production	*Cx32* KO model (Intraneural)	Ameliorated nerve pathology	[217]
	AAV9/*GJB1*or *CX32*	Cx32 production	*Cx32* KO model (Intrathecal)	Improved motor performance	[218]
	AAV1/*NT3*	Neurotrophic activity	*Cx32* KO model (Intramuscular)	Endured functional performance	[219]

Reference

1. Hertzog, N.; Jacob, C. Mechanisms and treatment strategies of demyelinating and dysmyelinating Charcot-Marie-Tooth disease. *Neural regeneration research* **2023**, *18*, 1931-1939, doi:10.4103/1673-5374.367834.
2. Li, J.; Parker, B.; Martyn, C.; Natarajan, C.; Guo, J. The PMP22 gene and its related diseases. *Molecular neurobiology* **2013**, *47*, 673-698, doi:10.1007/s12035-012-8370-x.

3. Bai, Y.; Wu, X.; Brennan, K.M.; Wang, D.S.; D'Antonio, M.; Moran, J.; Svaren, J.; Shy, M.E. Myelin protein zero mutations and the unfolded protein response in Charcot Marie Tooth disease type 1B. *Annals of clinical and translational neurology* **2018**, *5*, 445-455, doi:10.1002/acn3.543.
4. Bird, T.D. Historical perspective of defining Charcot-Marie-Tooth type 1B. *Annals of the New York Academy of Sciences* **1999**, *883*, 6-13.
5. Sanmaneechai, O.; Feely, S.; Scherer, S.S.; Herrmann, D.N.; Burns, J.; Muntoni, F.; Li, J.; Siskind, C.E.; Day, J.W.; Laura, M.; et al. Genotype-phenotype characteristics and baseline natural history of heritable neuropathies caused by mutations in the MPZ gene. *Brain : a journal of neurology* **2015**, *138*, 3180-3192, doi:10.1093/brain/awv241.
6. Caress, J.B.; Lewis, J.A.; Pinyan, C.W.; Lawson, V.H. A charcot-marie-tooth type 1B kindred associated with hemifacial spasm and trigeminal neuralgia. *Muscle & nerve* **2019**, *60*, 62-66, doi:10.1002/mus.26478.
7. Sanmaneechai, O.; Feely, S.; Scherer, S.S.; Herrmann, D.N.; Burns, J.; Muntoni, F.; Li, J.; Siskind, C.E.; Day, J.W.; Laura, M.; et al. Phenotype-genotype characteristics and baseline natural history of heritable neuropathies caused by mutations in the myelin protein zero gene. *Brain : a journal of neurology* **2015**, *138*, 3180-3192, doi:10.1093/brain/awv241.
8. Street, V.A.; Bennett, C.L.; Goldy, J.D.; Shirk, A.J.; Kleopa, K.A.; Tempel, B.L.; Lipe, H.P.; Scherer, S.S.; Bird, T.D.; Chance, P.F. Mutation of a putative protein degradation gene LITAF/SIMPLE in Charcot-Marie-Tooth disease 1C. *Neurology* **2003**, *60*, 22-26, doi:10.1212/wnl.60.1.22.
9. Park, J.; Kim, H.S.; Kwon, H.M.; Kim, J.; Nam, S.H.; Jung, N.Y.; Lee, A.J.; Jung, Y.H.; Kim, S.B.; Chung, K.W.; et al. Identification and clinical characterization of Charcot-Marie-Tooth disease type 1C patients with LITAF p.G112S mutation. *Genes & genomics* **2022**, *44*, 1007-1016, doi:10.1007/s13258-022-01253-w.
10. Echaniz-Laguna, A.; Cauquil, C.; Chanson, J.B.; Tard, C.; Guyant-Marechal, L.; Kuntzer, T.; Ion, I.M.; Lia, A.S.; Bouligand, J.; Poinsignon, V. EGR2 gene-linked hereditary neuropathies present with a bimodal age distribution at symptoms onset. *Journal of the peripheral nervous system : JPNS* **2023**, doi:10.1111/jns.12572.
11. Wang, D.S.; Wu, X.; Bai, Y.; Zaidman, C.; Grider, T.; Kamholz, J.; Lupski, J.R.; Connolly, A.M.; Shy, M.E. PMP22 exon 4 deletion causes ER retention of PMP22 and a gain-of-function allele in CMT1E. *Annals of clinical and translational neurology* **2017**, *4*, 236-245, doi:10.1002/acn3.395.
12. Taioli, F.; Bertolasi, L.; Ajena, D.; Ferrarini, M.; Cabrini, I.; Crestanello, A.; Fabrizi, G.M. Parental mosaicism of a novel PMP22 mutation with a minimal neuropathic phenotype. *Journal of the peripheral nervous system : JPNS* **2012**, *17*, 414-417, doi:10.1111/j.1529-8027.2012.00441.x.
13. Fernandes, M.; Caetano, A.; Castelhano, L.; Santos, L. Characterization of a Portuguese family with Charcot-Marie-Tooth disease type 1E due to a novel point mutation in the PMP22 gene. *Clinical neurology and neurosurgery* **2021**, *208*, 106829, doi:10.1016/j.clineuro.2021.106829.
14. Jung, N.Y.; Kwon, H.M.; Nam, D.E.; Tamanna, N.; Lee, A.J.; Kim, S.B.; Choi, B.O.; Chung, K.W. Peripheral Myelin Protein 22 Gene Mutations in Charcot-Marie-Tooth Disease Type 1E Patients. *Genes* **2022**, *13*, doi:10.3390/genes13071219.
15. Stone, E.J.; Kolb, S.J.; Brown, A. A review and analysis of the clinical literature on Charcot-Marie-Tooth disease caused by mutations in neurofilament protein L. *Cytoskeleton (Hoboken, N.J.)* **2021**, *78*, 97-110, doi:10.1002/cm.21676.
16. Shin, J.S.; Chung, K.W.; Cho, S.Y.; Yun, J.; Hwang, S.J.; Kang, S.H.; Cho, E.M.; Kim, S.M.; Choi, B.O. NEFL Pro22Arg mutation in Charcot-Marie-Tooth disease type 1. *Journal of human genetics* **2008**, *53*, 936-940, doi:10.1007/s10038-008-0333-8.
17. Hong, Y.B.; Joo, J.; Hyun, Y.S.; Kwak, G.; Choi, Y.R.; Yeo, H.K.; Jwa, D.H.; Kim, E.J.; Mo, W.M.; Nam, S.H.; et al. A Mutation in PMP2 Causes Dominant Demyelinating Charcot-Marie-Tooth Neuropathy. *PLoS genetics* **2016**, *12*, e1005829, doi:10.1371/journal.pgen.1005829.

18. Xu, F.; Takahashi, H.; Tanaka, Y.; Ichinose, S.; Niwa, S.; Wicklund, M.P.; Hirokawa, N. KIF1Bβ mutations detected in hereditary neuropathy impair IGF1R transport and axon growth. *The Journal of cell biology* **2018**, *217*, 3480-3496, doi:10.1083/jcb.201801085.
19. Saito, M.; Hayashi, Y.; Suzuki, T.; Tanaka, H.; Hozumi, I.; Tsuji, S. Linkage mapping of the gene for Charcot-Marie-Tooth disease type 2 to chromosome 1p (CMT2A) and the clinical features of CMT2A. *Neurology* **1997**, *49*, 1630-1635, doi:10.1212/wnl.49.6.1630.
20. Larrea, D.; Pera, M.; Gonnelli, A.; Quintana-Cabrera, R.; Akman, H.O.; Guardia-Laguarta, C.; Velasco, K.R.; Area-Gomez, E.; Dal Bello, F.; De Stefani, D.; et al. MFN2 mutations in Charcot-Marie-Tooth disease alter mitochondria-associated ER membrane function but do not impair bioenergetics. *Human molecular genetics* **2019**, *28*, 1782-1800, doi:10.1093/hmg/ddz008.
21. Muglia, M.; Zappia, M.; Timmerman, V.; Valentino, P.; Gabriele, A.L.; Conforti, F.L.; De Jonghe, P.; Ragno, M.; Mazzei, R.; Sabatelli, M.; et al. Clinical and genetic study of a large Charcot-Marie-Tooth type 2A family from southern Italy. *Neurology* **2001**, *56*, 100-103, doi:10.1212/wnl.56.1.100.
22. Lawson, V.H.; Graham, B.V.; Flanigan, K.M. Clinical and electrophysiologic features of CMT2A with mutations in the mitofusin 2 gene. *Neurology* **2005**, *65*, 197-204, doi:10.1212/01.wnl.0000168898.76071.70.
23. Cioni, J.M.; Lin, J.Q.; Holtermann, A.V.; Koppers, M.; Jakobs, M.A.H.; Azizi, A.; Turner-Bridger, B.; Shigeoka, T.; Franze, K.; Harris, W.A.; et al. Late Endosomes Act as mRNA Translation Platforms and Sustain Mitochondria in Axons. *Cell* **2019**, *176*, 56-72.e15, doi:10.1016/j.cell.2018.11.030.
24. Kwon, J.M.; Elliott, J.L.; Yee, W.C.; Ivanovich, J.; Scavarda, N.J.; Moolsintong, P.J.; Goodfellow, P.J. Assignment of a second Charcot-Marie-Tooth type II locus to chromosome 3q. *American journal of human genetics* **1995**, *57*, 853-858.
25. Houlden, H.; King, R.H.; Muddle, J.R.; Warner, T.T.; Reilly, M.M.; Orrell, R.W.; Ginsberg, L. A novel RAB7 mutation associated with ulcero-mutilating neuropathy. *Annals of neurology* **2004**, *56*, 586-590, doi:10.1002/ana.20281.
26. De Sandre-Giovannoli, A.; Chaouch, M.; Kozlov, S.; Vallat, J.M.; Tazir, M.; Kassouri, N.; Szepetowski, P.; Hammadouche, T.; Vandenberghe, A.; Stewart, C.L.; et al. Homozygous defects in LMNA, encoding lamin A/C nuclear-envelope proteins, cause autosomal recessive axonal neuropathy in human (Charcot-Marie-Tooth disorder type 2) and mouse. *American journal of human genetics* **2002**, *70*, 726-736, doi:10.1086/339274.
27. Lassuthová, P.; Baránková, L.; Haberlová, J.; Mazanec, R.; Wallace, A.; Huehne, K.; Rautenstrauss, B.; Seeman, P. Mutations in the LMNA gene do not cause axonal CMT in Czech patients. *Journal of human genetics* **2009**, *54*, 365-368, doi:10.1038/jhg.2009.43.
28. Bouhouche, A.; Benomar, A.; Birouk, N.; Mularoni, A.; Meggouh, F.; Tassin, J.; Grid, D.; Vandenberghe, A.; Yahyaoui, M.; Chkili, T.; et al. A locus for an axonal form of autosomal recessive Charcot-Marie-Tooth disease maps to chromosome 1q21.2-q21.3. *American journal of human genetics* **1999**, *65*, 722-727, doi:10.1086/302542.
29. Leal, A.; Bogantes-Ledezma, S.; Ekici, A.B.; Uebe, S.; Thiel, C.T.; Sticht, H.; Berghoff, M.; Berghoff, C.; Morera, B.; Meisterernst, M.; et al. The polynucleotide kinase 3'-phosphatase gene (PNKP) is involved in Charcot-Marie-Tooth disease (CMT2B2) previously related to MED25. *Neurogenetics* **2018**, *19*, 215-225, doi:10.1007/s10048-018-0555-7.
30. Pedroso, J.L.; Rocha, C.R.; Macedo-Souza, L.I.; De Mario, V.; Marques, W., Jr.; Barsottini, O.G.; Bulle Oliveira, A.S.; Menck, C.F.; Kok, F. Mutation in PNKP presenting initially as axonal Charcot-Marie-Tooth disease. *Neurology. Genetics* **2015**, *1*, e30, doi:10.1212/nxg.0000000000000030.
31. Leal, A.; Morera, B.; Del Valle, G.; Heuss, D.; Kayser, C.; Berghoff, M.; Villegas, R.; Hernández, E.; Méndez, M.; Hennies, H.C.; et al. A second locus for an axonal form of autosomal recessive Charcot-Marie-Tooth disease maps to chromosome 19q13.3. *American journal of human genetics* **2001**, *68*, 269-274, doi:10.1086/316934.

32. Klein, C.J.; Shi, Y.; Fecto, F.; Donaghy, M.; Nicholson, G.; McEntagart, M.E.; Crosby, A.H.; Wu, Y.; Lou, H.; McEvoy, K.M.; et al. TRPV4 mutations and cytotoxic hypercalcemia in axonal Charcot-Marie-Tooth neuropathies. *Neurology* **2011**, *76*, 887-894, doi:10.1212/WNL.0b013e31820f2de3.
33. Auer-Grumbach, M.; Olschewski, A.; Papić, L.; Kremer, H.; McEntagart, M.E.; Uhrig, S.; Fischer, C.; Fröhlich, E.; Bálint, Z.; Tang, B.; et al. Alterations in the ankyrin domain of TRPV4 cause congenital distal SMA, scapuloperoneal SMA and HMSN2C. *Nature genetics* **2010**, *42*, 160-164, doi:10.1038/ng.508.
34. Dyck, P.J.; Litchy, W.J.; Minnerath, S.; Bird, T.D.; Chance, P.F.; Schaid, D.J.; Aronson, A.E. Hereditary motor and sensory neuropathy with diaphragm and vocal cord paresis. *Annals of neurology* **1994**, *35*, 608-615, doi:10.1002/ana.410350515.
35. Donaghy, M.; Kennett, R. Varying occurrence of vocal cord paralysis in a family with autosomal dominant hereditary motor and sensory neuropathy. *Journal of neurology* **1999**, *246*, 552-555, doi:10.1007/s004150050402.
36. Chen, D.H.; Sul, Y.; Weiss, M.; Hillel, A.; Lipe, H.; Wolff, J.; Matsushita, M.; Raskind, W.; Bird, T. CMT2C with vocal cord paresis associated with short stature and mutations in the TRPV4 gene. *Neurology* **2010**, *75*, 1968-1975, doi:10.1212/WNL.0b013e3181ffe4bb.
37. Rebelo, A.P.; Abrams, A.J.; Cottenie, E.; Horga, A.; Gonzalez, M.; Bis, D.M.; Sanchez-Mejias, A.; Pinto, M.; Buglo, E.; Markel, K.; et al. Cryptic Amyloidogenic Elements in the 3' UTRs of Neurofilament Genes Trigger Axonal Neuropathy. *American journal of human genetics* **2016**, *98*, 597-614, doi:10.1016/j.ajhg.2016.02.022.
38. Abe, A.; Hayasaka, K. The GARS gene is rarely mutated in Japanese patients with Charcot-Marie-Tooth neuropathy. *Journal of human genetics* **2009**, *54*, 310-312, doi:10.1038/jhg.2009.25.
39. Antonellis, A.; Ellsworth, R.E.; Sambuughin, N.; Puls, I.; Abel, A.; Lee-Lin, S.Q.; Jordanova, A.; Kremensky, I.; Christodoulou, K.; Middleton, L.T.; et al. Glycyl tRNA synthetase mutations in Charcot-Marie-Tooth disease type 2D and distal spinal muscular atrophy type V. *American journal of human genetics* **2003**, *72*, 1293-1299, doi:10.1086/375039.
40. Sambuughin, N.; Sivakumar, K.; Selenge, B.; Lee, H.S.; Friedlich, D.; Baasanjav, D.; Dalakas, M.C.; Goldfarb, L.G. Autosomal dominant distal spinal muscular atrophy type V (dSMA-V) and Charcot-Marie-Tooth disease type 2D (CMT2D) segregate within a single large kindred and map to a refined region on chromosome 7p15. *Journal of the neurological sciences* **1998**, *161*, 23-28, doi:10.1016/s0022-510x(98)00264-0.
41. Ionasescu, V.; Searby, C.; Sheffield, V.C.; Roklina, T.; Nishimura, D.; Ionasescu, R. Autosomal dominant Charcot-Marie-Tooth axonal neuropathy mapped on chromosome 7p (CMT2D). *Human molecular genetics* **1996**, *5*, 1373-1375, doi:10.1093/hmg/5.9.1373.
42. Yalcouyé, A.; Diallo, S.H.; Coulibaly, T.; Cissé, L.; Diallo, S.; Samassékou, O.; Diarra, S.; Coulibaly, D.; Keita, M.; Guinto, C.O.; et al. A novel mutation in the GARS gene in a Malian family with Charcot-Marie-Tooth disease. *Molecular genetics & genomic medicine* **2019**, *7*, e00782, doi:10.1002/mgg3.782.
43. Lassuthova, P.; Rebelo, A.P.; Ravenscroft, G.; Lamont, P.J.; Davis, M.R.; Manganelli, F.; Feely, S.M.; Bacon, C.; Brožková, D.; Haberlova, J.; et al. Mutations in ATP1A1 cause dominant Charcot-Marie-Tooth type 2. *American journal of human genetics* **2018**, *102*, 505-514, doi:10.1016/j.ajhg.2018.01.023.
44. Zhai, J.; Lin, H.; Julien, J.P.; Schlaepfer, W.W. Disruption of neurofilament network with aggregation of light neurofilament protein: a common pathway leading to motor neuron degeneration due to Charcot-Marie-Tooth disease-linked mutations in NFL and HSPB1. *Human molecular genetics* **2007**, *16*, 3103-3116, doi:10.1093/hmg/ddm272.

45. Mersiyanova, I.V.; Perepelov, A.V.; Polyakov, A.V.; Sitnikov, V.F.; Dadali, E.L.; Oparin, R.B.; Petrin, A.N.; Evgrafov, O.V. A new variant of Charcot-Marie-Tooth disease type 2 is probably the result of a mutation in the neurofilament-light gene. *American journal of human genetics* **2000**, *67*, 37-46, doi:10.1086/302962.
46. Georgiou, D.M.; Zidar, J.; Korosec, M.; Middleton, L.T.; Kyriakides, T.; Christodoulou, K. A novel NF-L mutation Pro22Ser is associated with CMT2 in a large Slovenian family. *Neurogenetics* **2002**, *4*, 93-96, doi:10.1007/s10048-002-0138-4.
47. Miltenberger-Miltenyi, G.; Janecke, A.R.; Wanschitz, J.V.; Timmerman, V.; Windpassinger, C.; Auer-Grumbach, M.; Löscher, W.N. Clinical and electrophysiological features in Charcot-Marie-Tooth disease with mutations in the NEFL gene. *Archives of neurology* **2007**, *64*, 966-970, doi:10.1001/archneur.64.7.966.
48. Blakely, E.L.; Butterworth, A.; Hadden, R.D.; Bodi, I.; He, L.; McFarland, R.; Taylor, R.W. MPV17 mutation causes neuropathy and leukoencephalopathy with multiple mtDNA deletions in muscle. *Neuromuscular disorders : NMD* **2012**, *22*, 587-591, doi:10.1016/j.nmd.2012.03.006.
49. Baumann, M.; Schreiber, H.; Schlotter-Weigel, B.; Löscher, W.N.; Stucka, R.; Karall, D.; Strom, T.M.; Bauer, P.; Krabichler, B.; Fauth, C.; et al. MPV17 mutations in juvenile- and adult-onset axonal sensorimotor polyneuropathy. *Clinical genetics* **2019**, *95*, 182-186, doi:10.1111/cge.13462.
50. Ismailov, S.M.; Fedotov, V.P.; Dadali, E.L.; Polyakov, A.V.; Van Broeckhoven, C.; Ivanov, V.I.; De Jonghe, P.; Timmerman, V.; Evgrafov, O.V. A new locus for autosomal dominant Charcot-Marie-Tooth disease type 2 (CMT2F) maps to chromosome 7q11-q21. *European journal of human genetics : EJHG* **2001**, *9*, 646-650, doi:10.1038/sj.ejhg.5200686.
51. Tang, B.; Liu, X.; Zhao, G.; Luo, W.; Xia, K.; Pan, Q.; Cai, F.; Hu, Z.; Zhang, C.; Chen, B.; et al. Mutation analysis of the small heat shock protein 27 gene in chinese patients with Charcot-Marie-Tooth disease. *Archives of neurology* **2005**, *62*, 1201-1207, doi:10.1001/archneur.62.8.1201.
52. Rebelo, A.P.; Cortese, A.; Abraham, A.; Eshed-Eisenbach, Y.; Shner, G.; Vainshtein, A.; Buglo, E.; Camarena, V.; Gaidosh, G.; Shiekhattar, R.; et al. A CADM3 variant causes Charcot-Marie-Tooth disease with marked upper limb involvement. *Brain : a journal of neurology* **2021**, *144*, 1197-1213, doi:10.1093/brain/awab019.
53. Mendoza-Ferreira, N.; Karakaya, M.; Cengiz, N.; Beijer, D.; Brigatti, K.W.; Gonzaga-Jauregui, C.; Fuhrmann, N.; Hölker, I.; Thelen, M.P.; Zetzsche, S.; et al. De Novo and Inherited Variants in GBF1 are Associated with Axonal Neuropathy Caused by Golgi Fragmentation. *American journal of human genetics* **2020**, *107*, 763-777, doi:10.1016/j.ajhg.2020.08.018.
54. Niemann, A.; Ruegg, M.; La Padula, V.; Schenone, A.; Suter, U. Ganglioside-induced differentiation associated protein 1 is a regulator of the mitochondrial network: new implications for Charcot-Marie-Tooth disease. *The Journal of cell biology* **2005**, *170*, 1067-1078, doi:10.1083/jcb.200507087.
55. Niemann, A.; Wagner, K.M.; Ruegg, M.; Suter, U. GDAP1 mutations differ in their effects on mitochondrial dynamics and apoptosis depending on the mode of inheritance. *Neurobiology of disease* **2009**, *36*, 509-520, doi:10.1016/j.nbd.2009.09.011.
56. Barhoumi, C.; Amouri, R.; Ben Hamida, C.; Ben Hamida, M.; Machghoul, S.; Gueddiche, M.; Hentati, F. Linkage of a new locus for autosomal recessive axonal form of Charcot-Marie-Tooth disease to chromosome 8q21.3. *Neuromuscular disorders : NMD* **2001**, *11*, 27-34, doi:10.1016/s0960-8966(00)00162-0.

57. Sullivan, J.M.; Motley, W.W.; Johnson, J.O.; Aisenberg, W.H.; Marshall, K.L.; Barwick, K.E.; Kong, L.; Huh, J.S.; Saavedra-Rivera, P.C.; McEntagart, M.M.; et al. Dominant mutations of the Notch ligand Jagged1 cause peripheral neuropathy. *The Journal of clinical investigation* **2020**, *130*, 1506-1512, doi:10.1172/jci128152.
58. Boerkoel, C.F.; Takashima, H.; Garcia, C.A.; Olney, R.K.; Johnson, J.; Berry, K.; Russo, P.; Kennedy, S.; Teebi, A.S.; Scavina, M.; et al. Charcot-Marie-Tooth disease and related neuropathies: mutation distribution and genotype-phenotype correlation. *Annals of neurology* **2002**, *51*, 190-201, doi:10.1002/ana.10089.
59. Senderek, J.; Hermanns, B.; Lehmann, U.; Bergmann, C.; Marx, G.; Kabus, C.; Timmerman, V.; Stoltenburg-Didinger, G.; Schröder, J.M. Charcot-Marie-Tooth neuropathy type 2 and P0 point mutations: two novel amino acid substitutions (Asp61Gly; Tyr119Cys) and a possible "hotspot" on Thr124Met. *Brain pathology (Zurich, Switzerland)* **2000**, *10*, 235-248, doi:10.1111/j.1750-3639.2000.tb00257.x.
60. Kahle, K.T.; Flores, B.; Bharucha-Goebel, D.; Zhang, J.; Donkervoort, S.; Hegde, M.; Hussain, G.; Duran, D.; Liang, B.; Sun, D.; et al. Peripheral motor neuropathy is associated with defective kinase regulation of the KCC3 cotransporter. *Science signaling* **2016**, *9*, ra77, doi:10.1126/scisignal.aae0546.
61. Ando, M.; Higuchi, Y.; Yuan, J.; Yoshimura, A.; Taniguchi, T.; Takei, J.; Takeuchi, M.; Hiramatsu, Y.; Shimizu, F.; Kubota, M.; et al. Novel heterozygous variants of SLC12A6 in Japanese families with Charcot-Marie-Tooth disease. *Annals of clinical and translational neurology* **2022**, *9*, 902-911, doi:10.1002/acn3.51603.
62. Park, J.; Flores, B.R.; Scherer, K.; Kuepper, H.; Rossi, M.; Rupprich, K.; Rautenberg, M.; Deininger, N.; Weichselbaum, A.; Grimm, A.; et al. De novo variants in SLC12A6 cause sporadic early-onset progressive sensorimotor neuropathy. *Journal of medical genetics* **2020**, *57*, 283-288, doi:10.1136/jmedgenet-2019-106273.
63. De Jonghe, P.; Timmerman, V.; Ceuterick, C.; Nelis, E.; De Vriendt, E.; Löfgren, A.; Vercruyssen, A.; Verellen, C.; Van Maldergem, L.; Martin, J.J.; et al. The Thr124Met mutation in the peripheral myelin protein zero (MPZ) gene is associated with a clinically distinct Charcot-Marie-Tooth phenotype. *Brain : a journal of neurology* **1999**, *122 (Pt 2)*, 281-290, doi:10.1093/brain/122.2.281.
64. Kabzińska, D.; Korwin-Piotrowska, T.; Drechsler, H.; Drac, H.; Hausmanowa-Petrusewicz, I.; Kochański, A. Late-onset Charcot-Marie-Tooth type 2 disease with hearing impairment associated with a novel Pro105Thr mutation in the MPZ gene. *American journal of medical genetics. Part A* **2007**, *143a*, 2196-2199, doi:10.1002/ajmg.a.31908.
65. Zimoń, M.; Baets, J.; Fabrizi, G.M.; Jaakkola, E.; Kabzińska, D.; Pilch, J.; Schindler, A.B.; Cornblath, D.R.; Fischbeck, K.H.; Auer-Grumbach, M.; et al. Dominant GDAP1 mutations cause predominantly mild CMT phenotypes. *Neurology* **2011**, *77*, 540-548, doi:10.1212/WNL.0b013e318228fc70.
66. Birouk, N.; Azzedine, H.; Dubourg, O.; Muriel, M.P.; Benomar, A.; Hamadouche, T.; Maisonobe, T.; Ouazzani, R.; Brice, A.; Yahyaoui, M.; et al. Phenotypical features of a Moroccan family with autosomal recessive Charcot-Marie-Tooth disease associated with the S194X mutation in the GDAP1 gene. *Archives of neurology* **2003**, *60*, 598-604, doi:10.1001/archneur.60.4.598.
67. Carra, S.; Sivilotti, M.; Chávez Zobel, A.T.; Lambert, H.; Landry, J. HspB8, a small heat shock protein mutated in human neuromuscular disorders, has in vivo chaperone activity in cultured cells. *Human molecular genetics* **2005**, *14*, 1659-1669, doi:10.1093/hmg/ddi174.

68. Kang, K.H.; Han, J.E.; Kim, H.; Kim, S.; Hong, Y.B.; Yun, J.; Nam, S.H.; Choi, B.O.; Koh, H. PINK1 and Parkin Ameliorate the Loss of Motor Activity and Mitochondrial Dysfunction Induced by Peripheral Neuropathy-Associated HSPB8 Mutants in Drosophila Models. *Biomedicines* **2023**, *11*, doi:10.3390/biomedicines11030832.
69. Hinshaw, J.E. Dynamin and its role in membrane fission. *Annual review of cell and developmental biology* **2000**, *16*, 483-519, doi:10.1146/annurev.cellbio.16.1.483.
70. Fabrizi, G.M.; Ferrarini, M.; Cavallaro, T.; Cabrini, I.; Cerini, R.; Bertolasi, L.; Rizzuto, N. Two novel mutations in dynamin-2 cause axonal Charcot-Marie-Tooth disease. *Neurology* **2007**, *69*, 291-295, doi:10.1212/01.wnl.0000265820.51075.61.
71. Weterman, M.A.J.; Kuo, M.; Kenter, S.B.; Gordillo, S.; Karjosukarso, D.W.; Takase, R.; Bronk, M.; Oprescu, S.; van Ruissen, F.; Witteveen, R.J.W.; et al. Hypermorphic and hypomorphic AARS alleles in patients with CMT2N expand clinical and molecular heterogeneities. *Human molecular genetics* **2018**, *27*, 4036-4050, doi:10.1093/hmg/ddy290.
72. Hafezparast, M.; Klocke, R.; Ruhrberg, C.; Marquardt, A.; Ahmad-Annuar, A.; Bowen, S.; Lalli, G.; Witherden, A.S.; Hummerich, H.; Nicholson, S.; et al. Mutations in dynein link motor neuron degeneration to defects in retrograde transport. *Science (New York, N.Y.)* **2003**, *300*, 808-812, doi:10.1126/science.1083129.
73. Weedon, M.N.; Hastings, R.; Caswell, R.; Xie, W.; Paszkiewicz, K.; Antoniadi, T.; Williams, M.; King, C.; Greenhalgh, L.; Newbury-Ecob, R.; et al. Exome sequencing identifies a DYNC1H1 mutation in a large pedigree with dominant axonal Charcot-Marie-Tooth disease. *American journal of human genetics* **2011**, *89*, 308-312, doi:10.1016/j.ajhg.2011.07.002.
74. Weterman, M.A.; Sorrentino, V.; Kasher, P.R.; Jakobs, M.E.; van Engelen, B.G.; Fluiter, K.; de Wissel, M.B.; Sizarov, A.; Nürnberg, G.; Nürnberg, P.; et al. A frameshift mutation in LRSAM1 is responsible for a dominant hereditary polyneuropathy. *Human molecular genetics* **2012**, *21*, 358-370, doi:10.1093/hmg/ddr471.
75. Guernsey, D.L.; Jiang, H.; Bedard, K.; Evans, S.C.; Ferguson, M.; Matsuoka, M.; Macgillivray, C.; Nightingale, M.; Perry, S.; Rideout, A.L.; et al. Mutation in the gene encoding ubiquitin ligase LRSAM1 in patients with Charcot-Marie-Tooth disease. *PLoS genetics* **2010**, *6*, doi:10.1371/journal.pgen.1001081.
76. Xu, W.Y.; Gu, M.M.; Sun, L.H.; Guo, W.T.; Zhu, H.B.; Ma, J.F.; Yuan, W.T.; Kuang, Y.; Ji, B.J.; Wu, X.L.; et al. A nonsense mutation in DHTKD1 causes Charcot-Marie-Tooth disease type 2 in a large Chinese pedigree. *American journal of human genetics* **2012**, *91*, 1088-1094, doi:10.1016/j.ajhg.2012.09.018.
77. Balastik, M.; Ferraguti, F.; Pires-da Silva, A.; Lee, T.H.; Alvarez-Bolado, G.; Lu, K.P.; Gruss, P. Deficiency in ubiquitin ligase TRIM2 causes accumulation of neurofilament light chain and neurodegeneration. *Proceedings of the National Academy of Sciences of the United States of America* **2008**, *105*, 12016-12021, doi:10.1073/pnas.0802261105.
78. Ylikallio, E.; Pöyhönen, R.; Zimon, M.; De Vriendt, E.; Hilander, T.; Paetau, A.; Jordanova, A.; Lönnqvist, T.; Tyynismaa, H. Deficiency of the E3 ubiquitin ligase TRIM2 in early-onset axonal neuropathy. *Human molecular genetics* **2013**, *22*, 2975-2983, doi:10.1093/hmg/ddt149.
79. Pehlivan, D.; Coban Akdemir, Z.; Karaca, E.; Bayram, Y.; Jhangiani, S.; Yildiz, E.P.; Muzny, D.; Uluc, K.; Gibbs, R.A.; Elcioglu, N.; et al. Exome sequencing reveals homozygous TRIM2 mutation in a patient with early onset CMT and bilateral vocal cord paralysis. *Human genetics* **2015**, *134*, 671-673, doi:10.1007/s00439-015-1548-3.

80. Cottenie, E.; Kochanski, A.; Jordanova, A.; Bansagi, B.; Zimon, M.; Horga, A.; Jaunmuktane, Z.; Saveri, P.; Rasic, V.M.; Baets, J.; et al. Truncating and missense mutations in IGHMBP2 cause Charcot-Marie Tooth disease type 2. *American journal of human genetics* **2014**, *95*, 590-601, doi:10.1016/j.ajhg.2014.10.002.
81. Schottmann, G.; Jungbluth, H.; Schara, U.; Knierim, E.; Morales Gonzalez, S.; Gill, E.; Seifert, F.; Norwood, F.; Deshpande, C.; von Au, K.; et al. Recessive truncating IGHMBP2 mutations presenting as axonal sensorimotor neuropathy. *Neurology* **2015**, *84*, 523-531, doi:10.1212/wnl.0000000000001220.
82. Auer-Grumbach, M.; Toegel, S.; Schabhüttl, M.; Weinmann, D.; Chiari, C.; Bennett, D.L.H.; Beetz, C.; Klein, D.; Andersen, P.M.; Böhme, I.; et al. Rare Variants in MME, Encoding Metalloprotease Neprilysin, Are Linked to Late-Onset Autosomal-Dominant Axonal Polyneuropathies. *American journal of human genetics* **2016**, *99*, 607-623, doi:10.1016/j.ajhg.2016.07.008.
83. Higuchi, Y.; Hashiguchi, A.; Yuan, J.; Yoshimura, A.; Mitsui, J.; Ishiura, H.; Tanaka, M.; Ishihara, S.; Tanabe, H.; Nozuma, S.; et al. Mutations in MME cause an autosomal-recessive Charcot-Marie-Tooth disease type 2. *Annals of neurology* **2016**, *79*, 659-672, doi:10.1002/ana.24612.
84. Gonzalez, M.; McLaughlin, H.; Houlden, H.; Guo, M.; Yo-Tsen, L.; Hadjivassilious, M.; Speziani, F.; Yang, X.L.; Antonellis, A.; Reilly, M.M.; et al. Exome sequencing identifies a significant variant in methionyl-tRNA synthetase (MARS) in a family with late-onset CMT2. *Journal of neurology, neurosurgery, and psychiatry* **2013**, *84*, 1247-1249, doi:10.1136/jnnp-2013-305049.
85. Hyun, Y.S.; Park, H.J.; Heo, S.H.; Yoon, B.R.; Nam, S.H.; Kim, S.B.; Park, C.I.; Choi, B.O.; Chung, K.W. Rare variants in methionyl- and tyrosyl-tRNA synthetase genes in late-onset autosomal dominant Charcot-Marie-Tooth neuropathy. *Clinical genetics* **2014**, *86*, 592-594, doi:10.1111/cge.12327.
86. Tétreault, M.; Gonzalez, M.; Dicaire, M.J.; Allard, P.; Gehring, K.; Leblanc, D.; Leclerc, N.; Schondorf, R.; Mathieu, J.; Zuchner, S.; et al. Adult-onset painful axonal polyneuropathy caused by a dominant NAGLU mutation. *Brain : a journal of neurology* **2015**, *138*, 1477-1483, doi:10.1093/brain/awv074.
87. Vester, A.; Velez-Ruiz, G.; McLaughlin, H.M.; Lupski, J.R.; Talbot, K.; Vance, J.M.; Züchner, S.; Roda, R.H.; Fischbeck, K.H.; Biesecker, L.G.; et al. A loss-of-function variant in the human histidyl-tRNA synthetase (HARS) gene is neurotoxic in vivo. *Human mutation* **2013**, *34*, 191-199, doi:10.1002/humu.22210.
88. Montecchiani, C.; Pedace, L.; Lo Giudice, T.; Casella, A.; Mearini, M.; Gaudiello, F.; Pedroso, J.L.; Terracciano, C.; Caltagirone, C.; Massa, R.; et al. ALS5/SPG11/KIAA1840 mutations cause autosomal recessive axonal Charcot-Marie-Tooth disease. *Brain : a journal of neurology* **2016**, *139*, 73-85, doi:10.1093/brain/awv320.
89. Jerath, N.U.; Crockett, C.D.; Moore, S.A.; Shy, M.E.; Weihl, C.C.; Chou, T.F.; Grider, T.; Gonzalez, M.A.; Zuchner, S.; Swenson, A. Rare Manifestation of a c.290 C>T, p.Gly97Glu VCP Mutation. *Case reports in genetics* **2015**, *2015*, 239167, doi:10.1155/2015/239167.
90. Gonzalez, M.A.; Feely, S.M.; Speziani, F.; Strickland, A.V.; Danzi, M.; Bacon, C.; Lee, Y.; Chou, T.F.; Blanton, S.H.; Weihl, C.C.; et al. A novel mutation in VCP causes Charcot-Marie-Tooth Type 2 disease. *Brain : a journal of neurology* **2014**, *137*, 2897-2902, doi:10.1093/brain/awu224.
91. Albulym, O.M.; Kennerson, M.L.; Harms, M.B.; Drew, A.P.; Siddell, A.H.; Auer-Grumbach, M.; Pestronk, A.; Connolly, A.; Baloh, R.H.; Zuchner, S.; et al. MORC2 mutations cause axonal Charcot-Marie-Tooth disease with pyramidal signs. *Annals of neurology* **2016**, *79*, 419-427, doi:10.1002/ana.24575.

92. Zhu, D.; Kennerson, M.L.; Walizada, G.; Züchner, S.; Vance, J.M.; Nicholson, G.A. Charcot-Marie-Tooth with pyramidal signs is genetically heterogeneous: families with and without MFN2 mutations. *Neurology* **2005**, *65*, 496-497, doi:10.1212/01.wnl.0000171345.62270.29.
93. Hyun, Y.S.; Hong, Y.B.; Choi, B.O.; Chung, K.W. Clinico-genetics in Korean Charcot-Marie-Tooth disease type 2Z with MORC2 mutations. *Brain : a journal of neurology* **2016**, *139*, e40, doi:10.1093/brain/aww082.
94. Noack, R.; Frede, S.; Albrecht, P.; Henke, N.; Pfeiffer, A.; Knoll, K.; Dehmel, T.; Meyer Zu Hörste, G.; Stettner, M.; Kieseier, B.C.; et al. Charcot-Marie-Tooth disease CMT4A: GDAP1 increases cellular glutathione and the mitochondrial membrane potential. *Human molecular genetics* **2012**, *21*, 150-162, doi:10.1093/hmg/ddr450.
95. Ben Othmane, K.; Hentati, F.; Lennon, F.; Ben Hamida, C.; Blel, S.; Roses, A.D.; Pericak-Vance, M.A.; Ben Hamida, M.; Vance, J.M. Linkage of a locus (CMT4A) for autosomal recessive Charcot-Marie-Tooth disease to chromosome 8q. *Human molecular genetics* **1993**, *2*, 1625-1628, doi:10.1093/hmg/2.10.1625.
96. Wang, H.; Kaçar Bayram, A.; Sprute, R.; Ozdemir, O.; Cooper, E.; Pergande, M.; Efthymiou, S.; Nedic, I.; Mazaheri, N.; Stumpfe, K.; et al. Genotype-Phenotype Correlations in Charcot-Marie-Tooth Disease Due to MTMR2 Mutations and Implications in Membrane Trafficking. *Frontiers in neuroscience* **2019**, *13*, 974, doi:10.3389/fnins.2019.00974.
97. Senderek, J.; Bergmann, C.; Weber, S.; Ketelsen, U.P.; Schorle, H.; Rudnik-Schöneborn, S.; Büttner, R.; Buchheim, E.; Zerres, K. Mutation of the SBF2 gene, encoding a novel member of the myotubularin family, in Charcot-Marie-Tooth neuropathy type 4B2/11p15. *Human molecular genetics* **2003**, *12*, 349-356, doi:10.1093/hmg/ddg030.
98. Gambardella, A.; Bolino, A.; Muglia, M.; Valentino, P.; Bono, F.; Oliveri, R.L.; Sabatelli, M.; Brancolini, V.; Van Broeckhoven, C.; Romeo, G.; et al. Genetic heterogeneity in autosomal recessive hereditary motor and sensory neuropathy with focally folded myelin sheaths (CMT4B). *Neurology* **1998**, *50*, 799-801, doi:10.1212/wnl.50.3.799.
99. Flusser, H.; Halperin, D.; Kadir, R.; Shorer, Z.; Shelef, I.; Birk, O.S. Novel SBF1 splice-site null mutation broadens the clinical spectrum of Charcot-Marie-Tooth type 4B3 disease. *Clinical genetics* **2018**, *94*, 473-479, doi:10.1111/cge.13419.
100. Alazami, A.M.; Alzahrani, F.; Bohlega, S.; Alkuraya, F.S. SET binding factor 1 (SBF1) mutation causes Charcot-Marie-tooth disease type 4B3. *Neurology* **2014**, *82*, 1665-1666, doi:10.1212/wnl.0000000000000331.
101. Bohlega, S.; Alazami, A.M.; Cupler, E.; Al-Hindi, H.; Ibrahim, E.; Alkuraya, F.S. A novel syndromic form of sensory-motor polyneuropathy is linked to chromosome 22q13.31-q13.33. *Clinical genetics* **2011**, *79*, 193-195, doi:10.1111/j.1399-0004.2010.01524.x.
102. Piscosquito, G.; Saveri, P.; Magri, S.; Ciano, C.; Gandioli, C.; Morbin, M.; Bella, D.D.; Moroni, I.; Taroni, F.; Pareyson, D. Screening for SH3TC2 gene mutations in a series of demyelinating recessive Charcot-Marie-Tooth disease (CMT4). *Journal of the peripheral nervous system : JPNS* **2016**, *21*, 142-149, doi:10.1111/jns.12175.
103. Colomer, J.; Gooding, R.; Angelicheva, D.; King, R.H.; Guillén-Navarro, E.; Parman, Y.; Nascimento, A.; Conill, J.; Kalaydjieva, L. Clinical spectrum of CMT4C disease in patients homozygous for the p.Arg1109X mutation in SH3TC2. *Neuromuscular disorders : NMD* **2006**, *16*, 449-453, doi:10.1016/j.nmd.2006.05.005.

104. Gabreëls-Festen, A.; van Beersum, S.; Eshuis, L.; LeGuern, E.; Gabreëls, F.; van Engelen, B.; Mariman, E. Study on the gene and phenotypic characterisation of autosomal recessive demyelinating motor and sensory neuropathy (Charcot-Marie-Tooth disease) with a gene locus on chromosome 5q23-q33. *Journal of neurology, neurosurgery, and psychiatry* **1999**, *66*, 569-574, doi:10.1136/jnnp.66.5.569.
105. Azzedine, H.; Ravisé, N.; Verny, C.; Gabrëels-Festen, A.; Lammens, M.; Grid, D.; Vallat, J.M.; Durosier, G.; Senderek, J.; Nouioua, S.; et al. Spine deformities in Charcot-Marie-Tooth 4C caused by SH3TC2 gene mutations. *Neurology* **2006**, *67*, 602-606, doi:10.1212/01.wnl.0000230225.19797.93.
106. Okuda, T.; Higashi, Y.; Kokame, K.; Tanaka, C.; Kondoh, H.; Miyata, T. Ndrg1-deficient mice exhibit a progressive demyelinating disorder of peripheral nerves. *Molecular and cellular biology* **2004**, *24*, 3949-3956, doi:10.1128/mcb.24.9.3949-3956.2004.
107. Ricard, E.; Mathis, S.; Magdelaine, C.; Delisle, M.B.; Magy, L.; Funalot, B.; Vallat, J.M. CMT4D (NDRG1 mutation): genotype-phenotype correlations. *Journal of the peripheral nervous system : JPNS* **2013**, *18*, 261-265, doi:10.1111/jns5.12039.
108. Okamoto, Y.; Goksungur, M.T.; Pehlivan, D.; Beck, C.R.; Gonzaga-Jauregui, C.; Muzny, D.M.; Atik, M.M.; Carvalho, C.M.B.; Matur, Z.; Bayraktar, S.; et al. Exonic duplication CNV of NDRG1 associated with autosomal-recessive HMSN-Lom/CMT4D. *Genetics in medicine : official journal of the American College of Medical Genetics* **2014**, *16*, 386-394, doi:10.1038/gim.2013.155.
109. Merlini, L.; Villanova, M.; Sabatelli, P.; Trogu, A.; Malandrini, A.; Yanakiev, P.; Maraldi, N.M.; Kalaydjieva, L. Hereditary motor and sensory neuropathy Lom type in an Italian Gypsy family. *Neuromuscular disorders : NMD* **1998**, *8*, 182-185, doi:10.1016/s0960-8966(98)00023-6.
110. Kalaydjieva, L.; Hallmayer, J.; Chandler, D.; Savov, A.; Nikolova, A.; Angelicheva, D.; King, R.H.; Ishpekova, B.; Honeyman, K.; Calafell, F.; et al. Gene mapping in Gypsies identifies a novel demyelinating neuropathy on chromosome 8q24. *Nature genetics* **1996**, *14*, 214-217, doi:10.1038/ng1096-214.
111. Warner, L.E.; Mancias, P.; Butler, I.J.; McDonald, C.M.; Keppen, L.; Koob, K.G.; Lupski, J.R. Mutations in the early growth response 2 (EGR2) gene are associated with hereditary myelinopathies. *Nature genetics* **1998**, *18*, 382-384, doi:10.1038/ng0498-382.
112. Tokunaga, S.; Hashiguchi, A.; Yoshimura, A.; Maeda, K.; Suzuki, T.; Haruki, H.; Nakamura, T.; Okamoto, Y.; Takashima, H. Late-onset Charcot-Marie-Tooth disease 4F caused by periaxin gene mutation. *Neurogenetics* **2012**, *13*, 359-365, doi:10.1007/s10048-012-0338-5.
113. Boerkoel, C.F.; Takashima, H.; Stankiewicz, P.; Garcia, C.A.; Leber, S.M.; Rhee-Morris, L.; Lupski, J.R. Periaxin mutations cause recessive Dejerine-Sottas neuropathy. *American journal of human genetics* **2001**, *68*, 325-333, doi:10.1086/318208.
114. Guilbot, A.; Williams, A.; Ravisé, N.; Verny, C.; Brice, A.; Sherman, D.L.; Brophy, P.J.; LeGuern, E.; Delague, V.; Bareil, C.; et al. A mutation in periaxin is responsible for CMT4F, an autosomal recessive form of Charcot-Marie-Tooth disease. *Human molecular genetics* **2001**, *10*, 415-421, doi:10.1093/hmg/10.4.415.
115. Takashima, H.; Boerkoel, C.F.; De Jonghe, P.; Ceuterick, C.; Martin, J.J.; Voit, T.; Schröder, J.M.; Williams, A.; Brophy, P.J.; Timmerman, V.; et al. Periaxin mutations cause a broad spectrum of demyelinating neuropathies. *Annals of neurology* **2002**, *51*, 709-715, doi:10.1002/ana.10213.
116. Marchesi, C.; Milani, M.; Morbin, M.; Cesani, M.; Lauria, G.; Scaioli, V.; Piccolo, G.; Fabrizi, G.M.; Cavallaro, T.; Taroni, F.; et al. Four novel cases of periaxin-related neuropathy and review of the literature. *Neurology* **2010**, *75*, 1830-1838, doi:10.1212/WNL.0b013e3181fd6314.
117. Kabzinska, D.; Drac, H.; Sherman, D.L.; Kostera-Pruszczyk, A.; Brophy, P.J.; Kochanski, A.; Hausmanowa-Petrusewicz, I. Charcot-Marie-Tooth type 4F disease caused by S399fsx410 mutation in the PRX gene. *Neurology* **2006**, *66*, 745-747, doi:10.1212/01.wnl.0000201269.46071.35.

118. Stendel, C.; Roos, A.; Deconinck, T.; Pereira, J.; Castagner, F.; Niemann, A.; Kirschner, J.; Korinthenberg, R.; Ketelsen, U.P.; Battaloglu, E.; et al. Peripheral nerve demyelination caused by a mutant Rho GTPase guanine nucleotide exchange factor, frabin/FGD4. *American journal of human genetics* **2007**, *81*, 158-164, doi:10.1086/518770.
119. Fabrizi, G.M.; Taioli, F.; Cavallaro, T.; Ferrari, S.; Bertolasi, L.; Casarotto, M.; Rizzuto, N.; Deconinck, T.; Timmerman, V.; De Jonghe, P. Further evidence that mutations in FGD4/frabin cause Charcot-Marie-Tooth disease type 4H. *Neurology* **2009**, *72*, 1160-1164, doi:10.1212/01.wnl.0000345373.58618.b6.
120. Houlden, H.; Hammans, S.; Katifi, H.; Reilly, M.M. A novel Frabin (FGD4) nonsense mutation p.R275X associated with phenotypic variability in CMT4H. *Neurology* **2009**, *72*, 617-620, doi:10.1212/01.wnl.0000342463.35089.cc.
121. Nicholson, G.; Lenk, G.M.; Reddel, S.W.; Grant, A.E.; Towne, C.F.; Ferguson, C.J.; Simpson, E.; Scheuerle, A.; Yasick, M.; Hoffman, S.; et al. Distinctive genetic and clinical features of CMT4J: a severe neuropathy caused by mutations in the PI(3,5)P_2 phosphatase FIG4. *Brain : a journal of neurology* **2011**, *134*, 1959-1971, doi:10.1093/brain/awr148.
122. Ferguson, C.J.; Lenk, G.M.; Meisler, M.H. Defective autophagy in neurons and astrocytes from mice deficient in PI(3,5)P2. *Human molecular genetics* **2009**, *18*, 4868-4878, doi:10.1093/hmg/ddp460.
123. Cottenie, E.; Menezes, M.P.; Rossor, A.M.; Morrow, J.M.; Yousry, T.A.; Dick, D.J.; Anderson, J.R.; Jaunmuktane, Z.; Brandner, S.; Blake, J.C.; et al. Rapidly progressive asymmetrical weakness in Charcot-Marie-Tooth disease type 4J resembles chronic inflammatory demyelinating polyneuropathy. *Neuromuscular disorders : NMD* **2013**, *23*, 399-403, doi:10.1016/j.nmd.2013.01.010.
124. Menezes, M.P.; Waddell, L.; Lenk, G.M.; Kaur, S.; MacArthur, D.G.; Meisler, M.H.; Clarke, N.F. Whole exome sequencing identifies three recessive FIG4 mutations in an apparently dominant pedigree with Charcot-Marie-Tooth disease. *Neuromuscular disorders : NMD* **2014**, *24*, 666-670, doi:10.1016/j.nmd.2014.04.010.
125. Dell'agnello, C.; Leo, S.; Agostino, A.; Szabadkai, G.; Tiveron, C.; Zulian, A.; Prelle, A.; Roubertoux, P.; Rizzuto, R.; Zeviani, M. Increased longevity and refractoriness to Ca(2+)-dependent neurodegeneration in Surf1 knockout mice. *Human molecular genetics* **2007**, *16*, 431-444, doi:10.1093/hmg/ddl477.
126. Deepa, S.S.; Pulliam, D.; Hill, S.; Shi, Y.; Walsh, M.E.; Salmon, A.; Sloane, L.; Zhang, N.; Zeviani, M.; Viscomi, C.; et al. Improved insulin sensitivity associated with reduced mitochondrial complex IV assembly and activity. *FASEB journal : official publication of the Federation of American Societies for Experimental Biology* **2013**, *27*, 1371-1380, doi:10.1096/fj.12-221879.
127. Echaniz-Laguna, A.; Ghezzi, D.; Chassagne, M.; Mayençon, M.; Padet, S.; Melchionda, L.; Rouvet, I.; Lannes, B.; Bozon, D.; Latour, P.; et al. SURF1 deficiency causes demyelinating Charcot-Marie-Tooth disease. *Neurology* **2013**, *81*, 1523-1530, doi:10.1212/WNL.0b013e3182a4a518.
128. Spira, P.J.; McLeod, J.G.; Evans, W.A. A spinocerebellar degeneration with X-linked inheritance. *Brain : a journal of neurology* **1979**, *102*, 27-41, doi:10.1093/brain/102.1.27.
129. Yiu, E.M.; Geevasinga, N.; Nicholson, G.A.; Fagan, E.R.; Ryan, M.M.; Ouvrier, R.A. A retrospective review of X-linked Charcot-Marie-Tooth disease in childhood. *Neurology* **2011**, *76*, 461-466, doi:10.1212/WNL.0b013e31820a0ceb.
130. Zhao, Y.; Xie, Y.; Zhu, X.; Wang, H.; Li, Y.; Li, J. Transient, recurrent, white matter lesions in x-linked Charcot-Marie-tooth disease with novel mutation of gap junction protein beta 1 gene in China: a case report. *BMC neurology* **2014**, *14*, 156, doi:10.1186/s12883-014-0156-5.

131. Ionasescu, V.V. Charcot-Marie-Tooth neuropathies: from clinical description to molecular genetics. *Muscle & nerve* **1995**, *18*, 267-275, doi:10.1002/mus.880180302.
132. Brewer, M.; Changi, F.; Antonellis, A.; Fischbeck, K.; Polly, P.; Nicholson, G.; Kennerson, M. Evidence of a founder haplotype refines the X-linked Charcot-Marie-Tooth (CMTX3) locus to a 2.5 Mb region. *Neurogenetics* **2008**, *9*, 191-195, doi:10.1007/s10048-008-0126-4.
133. Chaudhry, R.; Kidambi, A.; Brewer, M.H.; Antonellis, A.; Mathews, K.; Nicholson, G.; Kennerson, M. Re-analysis of an original CMTX3 family using exome sequencing identifies a known BSCL2 mutation. *Muscle & nerve* **2013**, *47*, 922-924, doi:10.1002/mus.23743.
134. Kanhangad, M.; Cornett, K.; Brewer, M.H.; Nicholson, G.A.; Ryan, M.M.; Smith, R.L.; Subramanian, G.M.; Young, H.K.; Züchner, S.; Kennerson, M.L.; et al. Unique clinical and neurophysiologic profile of a cohort of children with CMTX3. *Neurology* **2018**, *90*, e1706-e1710, doi:10.1212/wnl.0000000000005479.
135. Wang, B.; Li, X.; Wang, J.; Liu, L.; Xie, Y.; Huang, S.; Pakhrin, P.S.; Jin, Q.; Zhu, C.; Tang, B.; et al. A novel AIFM1 mutation in a Chinese family with X-linked Charcot-Marie-Tooth disease type 4. *Neuromuscular disorders : NMD* **2018**, *28*, 652-659, doi:10.1016/j.nmd.2018.05.008.
136. Diodato, D.; Tasca, G.; Verrigni, D.; D'Amico, A.; Rizza, T.; Tozzi, G.; Martinelli, D.; Verardo, M.; Invernizzi, F.; Nasca, A.; et al. A novel AIFM1 mutation expands the phenotype to an infantile motor neuron disease. *European journal of human genetics : EJHG* **2016**, *24*, 463-466, doi:10.1038/ejhg.2015.141.
137. Rinaldi, C.; Grunseich, C.; Sevrioukova, I.F.; Schindler, A.; Horkayne-Szakaly, I.; Lamperti, C.; Landouré, G.; Kennerson, M.L.; Burnett, B.G.; Bönnemann, C.; et al. Cowchock syndrome is associated with a mutation in apoptosis-inducing factor. *American journal of human genetics* **2012**, *91*, 1095-1102, doi:10.1016/j.ajhg.2012.10.008.
138. Ardissone, A.; Piscosquito, G.; Legati, A.; Langella, T.; Lamantea, E.; Garavaglia, B.; Salsano, E.; Farina, L.; Moroni, I.; Pareyson, D.; et al. A slowly progressive mitochondrial encephalomyopathy widens the spectrum of AIFM1 disorders. *Neurology* **2015**, *84*, 2193-2195, doi:10.1212/wnl.0000000000001613.
139. Heimer, G.; Eyal, E.; Zhu, X.; Ruzzo, E.K.; Marek-Yagel, D.; Sagiv, D.; Anikster, Y.; Reznik-Wolf, H.; Pras, E.; Oz Levi, D.; et al. Mutations in AIFM1 cause an X-linked childhood cerebellar ataxia partially responsive to riboflavin. *European journal of paediatric neurology : EJPN : official journal of the European Paediatric Neurology Society* **2018**, *22*, 93-101, doi:10.1016/j.ejpn.2017.09.004.
140. Bogdanova-Mihaylova, P.; Alexander, M.D.; Murphy, R.P.; Chen, H.; Healy, D.G.; Walsh, R.A.; Murphy, S.M. Clinical spectrum of AIFM1-associated disease in an Irish family, from mild neuropathy to severe cerebellar ataxia with colour blindness. *Journal of the peripheral nervous system : JPNS* **2019**, *24*, 348-353, doi:10.1111/jns.12348.
141. Kim, H.J.; Sohn, K.M.; Shy, M.E.; Krajewski, K.M.; Hwang, M.; Park, J.H.; Jang, S.Y.; Won, H.H.; Choi, B.O.; Hong, S.H.; et al. Mutations in PRPS1, which encodes the phosphoribosyl pyrophosphate synthetase enzyme critical for nucleotide biosynthesis, cause hereditary peripheral neuropathy with hearing loss and optic neuropathy (cmtx5). *American journal of human genetics* **2007**, *81*, 552-558, doi:10.1086/519529.
142. Rosenberg, R.N.; Chutorian, A. Familial opticoacoustic nerve degeneration and polyneuropathy. *Neurology* **1967**, *17*, 827-832, doi:10.1212/wnl.17.9.827.
143. Kim, H.J.; Hong, S.H.; Ki, C.S.; Kim, B.J.; Shim, J.S.; Cho, S.H.; Park, J.H.; Kim, J.W. A novel locus for X-linked recessive CMT with deafness and optic neuropathy maps to Xq21.32-q24. *Neurology* **2005**, *64*, 1964-1967, doi:10.1212/01.Wnl.0000163768.58168.3a.

144. Park, J.; Hyun, Y.S.; Kim, Y.J.; Nam, S.H.; Kim, S.H.; Hong, Y.B.; Park, J.M.; Chung, K.W.; Choi, B.O. Exome Sequencing Reveals a Novel PRPS1 Mutation in a Family with CMTX5 without Optic Atrophy. *Journal of clinical neurology (Seoul, Korea)* **2013**, *9*, 283-288, doi:10.3988/jcn.2013.9.4.283.
145. Almoguera, B.; He, S.; Corton, M.; Fernandez-San Jose, P.; Blanco-Kelly, F.; López-Molina, M.I.; García-Sandoval, B.; Del Val, J.; Guo, Y.; Tian, L.; et al. Expanding the phenotype of PRPS1 syndromes in females: neuropathy, hearing loss and retinopathy. *Orphanet journal of rare diseases* **2014**, *9*, 190, doi:10.1186/s13023-014-0190-9.
146. Kennerson, M.L.; Yiu, E.M.; Chuang, D.T.; Kidambi, A.; Tso, S.C.; Ly, C.; Chaudhry, R.; Drew, A.P.; Rance, G.; Delatycki, M.B.; et al. A new locus for X-linked dominant Charcot-Marie-Tooth disease (CMTX6) is caused by mutations in the pyruvate dehydrogenase kinase isoenzyme 3 (PDK3) gene. *Human molecular genetics* **2013**, *22*, 1404-1416, doi:10.1093/hmg/dds557.
147. Chetlin, R.D.; Gutmann, L.; Tarnopolsky, M.; Ullrich, I.H.; Yeater, R.A. Resistance training effectiveness in patients with Charcot-Marie-Tooth disease: recommendations for exercise prescription. *Archives of physical medicine and rehabilitation* **2004**, *85*, 1217-1223, doi:10.1016/j.apmr.2003.12.025.
148. Chetlin, R.D.; Gutmann, L.; Tarnopolsky, M.A.; Ullrich, I.H.; Yeater, R.A. Resistance training exercise and creatine in patients with Charcot-Marie-Tooth disease. *Muscle & nerve* **2004**, *30*, 69-76, doi:10.1002/mus.20078.
149. Ramdharry, G.M.; Pollard, A.; Anderson, C.; Laurá, M.; Murphy, S.M.; Dudziec, M.; Dewar, E.L.; Hutton, E.; Grant, R.; Reilly, M.M. A pilot study of proximal strength training in Charcot-Marie-Tooth disease. *Journal of the peripheral nervous system : JPNS* **2014**, *19*, 328-332, doi:10.1111/jns.12100.
150. Burns, J.; Raymond, J.; Ouvrier, R. Feasibility of foot and ankle strength training in childhood Charcot-Marie-Tooth disease. *Neuromuscular disorders : NMD* **2009**, *19*, 818-821, doi:10.1016/j.nmd.2009.09.007.
151. Burns, J.; Sman, A.D.; Cornett, K.M.D.; Wojciechowski, E.; Walker, T.; Menezes, M.P.; Mandarakas, M.R.; Rose, K.J.; Bray, P.; Sampaio, H.; et al. Safety and efficacy of progressive resistance exercise for Charcot-Marie-Tooth disease in children: a randomised, double-blind, sham-controlled trial. *The Lancet. Child & adolescent health* **2017**, *1*, 106-113, doi:10.1016/s2352-4642(17)30013-5.
152. Voet, N.B.; van der Kooi, E.L.; Riphagen, II; Lindeman, E.; van Engelen, B.G.; Geurts, A.C. Strength training and aerobic exercise training for muscle disease. *The Cochrane database of systematic reviews* **2013**, Cd003907, doi:10.1002/14651858.CD003907.pub4.
153. Lindeman, E.; Leffers, P.; Spaans, F.; Drukker, J.; Reulen, J.; Kerckhoffs, M.; Köke, A. Strength training in patients with myotonic dystrophy and hereditary motor and sensory neuropathy: a randomized clinical trial. *Archives of physical medicine and rehabilitation* **1995**, *76*, 612-620, doi:10.1016/s0003-9993(95)80629-6.
154. Lindeman, E.; Spaans, F.; Reulen, J.; Leffers, P.; Drukker, J. Progressive resistance training in neuromuscular patients. Effects on force and surface EMG. *Journal of electromyography and kinesiology : official journal of the International Society of Electrophysiological Kinesiology* **1999**, *9*, 379-384, doi:10.1016/s1050-6411(99)00003-6.
155. Florence, J.M.; Hagberg, J.M. Effect of training on the exercise responses of neuromuscular disease patients. *Medicine and science in sports and exercise* **1984**, *16*, 460-465, doi:10.1249/00005768-198410000-00007.
156. El Mhandi, L.; Millet, G.Y.; Calmels, P.; Richard, A.; Oullion, R.; Gautheron, V.; Féasson, L. Benefits of interval-training on fatigue and functional capacities in Charcot-Marie-Tooth disease. *Muscle & nerve* **2008**, *37*, 601-610, doi:10.1002/mus.20959.

157. Wright, N.C.; Kilmer, D.D.; McCrory, M.A.; Aitkens, S.G.; Holcomb, B.J.; Bernauer, E.M. Aerobic walking in slowly progressive neuromuscular disease: effect of a 12-week program. *Archives of physical medicine and rehabilitation* **1996**, *77*, 64-69, doi:10.1016/s0003-9993(96)90222-1.
158. Maggi, G.; Monti Bragadin, M.; Padua, L.; Fiorina, E.; Bellone, E.; Grandis, M.; Reni, L.; Bennicelli, A.; Grosso, M.; Saporiti, R.; et al. Outcome measures and rehabilitation treatment in patients affected by Charcot-Marie-Tooth neuropathy: a pilot study. *American journal of physical medicine & rehabilitation* **2011**, *90*, 628-637, doi:10.1097/PHM.0b013e31821f6e32.
159. Mori, L.; Signori, A.; Prada, V.; Pareyson, D.; Piscosquito, G.; Padua, L.; Pazzaglia, C.; Fabrizi, G.M.; Picelli, A.; Schenone, A. Treadmill training in patients affected by Charcot-Marie-Tooth neuropathy: results of a multicenter, prospective, randomized, single-blind, controlled study. *European journal of neurology* **2020**, *27*, 280-287, doi:10.1111/ene.14074.
160. Matjacić, Z.; Zupan, A. Effects of dynamic balance training during standing and stepping in patients with hereditary sensory motor neuropathy. *Disability and rehabilitation* **2006**, *28*, 1455-1459, doi:10.1080/09638280600646169.
161. Prukop, T.; Stenzel, J.; Wernick, S.; Kungl, T.; Mroczek, M.; Adam, J.; Ewers, D.; Nabirotchkin, S.; Nave, K.A.; Hajj, R.; et al. Early short-term PXT3003 combinational therapy delays disease onset in a transgenic rat model of Charcot-Marie-Tooth disease 1A (CMT1A). *PLoS One* **2019**, *14*, e0209752, doi:10.1371/journal.pone.0209752.
162. Prukop, T.; Wernick, S.; Boussicault, L.; Ewers, D.; Jäger, K.; Adam, J.; Winter, L.; Quintes, S.; Linhoff, L.; Barrantes-Freer, A.; et al. Synergistic PXT3003 therapy uncouples neuromuscular function from dysmyelination in male Charcot-Marie-Tooth disease type 1A (CMT1A) rats. *Journal of neuroscience research* **2020**, *98*, 1933-1952, doi:10.1002/jnr.24679.
163. Meyer zu Horste, G.; Prukop, T.; Liebetanz, D.; Mobius, W.; Nave, K.A.; Sereda, M.W. Antiprogesterone therapy uncouples axonal loss from demyelination in a transgenic rat model of CMT1A neuropathy. *Annals of neurology* **2007**, *61*, 61-72, doi:10.1002/ana.21026.
164. Sereda, M.W.; Meyer zu Hörste, G.; Suter, U.; Uzma, N.; Nave, K.A. Therapeutic administration of progesterone antagonist in a model of Charcot-Marie-Tooth disease (CMT-1A). *Nature medicine* **2003**, *9*, 1533-1537, doi:10.1038/nm957.
165. Passage, E.; Norreel, J.C.; Noack-Fraissignes, P.; Sanguedolce, V.; Pizant, J.; Thirion, X.; Robaglia-Schlupp, A.; Pellissier, J.F.; Fontés, M. Ascorbic acid treatment corrects the phenotype of a mouse model of Charcot-Marie-Tooth disease. *Nature medicine* **2004**, *10*, 396-401, doi:10.1038/nm1023.
166. Verhamme, C.; de Haan, R.J.; Vermeulen, M.; Baas, F.; de Visser, M.; van Schaik, I.N. Oral high dose ascorbic acid treatment for one year in young CMT1A patients: a randomised, double-blind, placebo-controlled phase II trial. *BMC medicine* **2009**, *7*, 70, doi:10.1186/1741-7015-7-70.
167. Madorsky, I.; Opalach, K.; Waber, A.; Verrier, J.D.; Solmo, C.; Foster, T.; Dunn, W.A., Jr.; Notterpek, L. Intermittent fasting alleviates the neuropathic phenotype in a mouse model of Charcot-Marie-Tooth disease. *Neurobiology of disease* **2009**, *34*, 146-154, doi:10.1016/j.nbd.2009.01.002.
168. Chittoor-Vinod, V.G.; Bazick, H.; Todd, A.G.; Falk, D.; Morelli, K.H.; Burgess, R.W.; Foster, T.C.; Notterpek, L. HSP90 Inhibitor, NVP-AUY922, Improves Myelination in Vitro and Supports the Maintenance of Myelinated Axons in Neuropathic Mice. *ACS chemical neuroscience* **2019**, *10*, 2890-2902, doi:10.1021/acschemneuro.9b00105.
169. Nobbio, L.; Sturla, L.; Fiorese, F.; Usai, C.; Basile, G.; Moreschi, I.; Benvenuto, F.; Zocchi, E.; De Flora, A.; Schenone, A.; et al. P2X7-mediated increased intracellular calcium causes functional derangement in Schwann cells from rats with CMT1A neuropathy. *The Journal of biological chemistry* **2009**, *284*, 23146-23158, doi:10.1074/jbc.M109.027128.

170. Sociali, G.; Visigalli, D.; Prukop, T.; Cervellini, I.; Mannino, E.; Venturi, C.; Bruzzone, S.; Sereda, M.W.; Schenone, A. Tolerability and efficacy study of P2X7 inhibition in experimental Charcot-Marie-Tooth type 1A (CMT1A) neuropathy. *Neurobiology of disease* **2016**, *95*, 145-157, doi:10.1016/j.nbd.2016.07.017.
171. Fledrich, R.; Stassart, R.M.; Klink, A.; Rasch, L.M.; Prukop, T.; Haag, L.; Czesnik, D.; Kungl, T.; Abdelaal, T.A.; Keric, N.; et al. Soluble neuregulin-1 modulates disease pathogenesis in rodent models of Charcot-Marie-Tooth disease 1A. *Nature medicine* **2014**, *20*, 1055-1061, doi:10.1038/nm.3664.
172. Fledrich, R.; Abdelaal, T.; Rasch, L.; Bansal, V.; Schütza, V.; Brügger, B.; Lüchtenborg, C.; Prukop, T.; Stenzel, J.; Rahman, R.U.; et al. Targeting myelin lipid metabolism as a potential therapeutic strategy in a model of CMT1A neuropathy. *Nature communications* **2018**, *9*, 3025, doi:10.1038/s41467-018-05420-0.
173. Caillaud, M.; Msheik, Z.; Ndong-Ntoutoume, G.M.; Vignaud, L.; Richard, L.; Favreau, F.; Faye, P.A.; Sturtz, F.; Granet, R.; Vallat, J.M.; et al. Curcumin-cyclodextrin/cellulose nanocrystals improve the phenotype of Charcot-Marie-Tooth-1A transgenic rats through the reduction of oxidative stress. *Free radical biology & medicine* **2020**, *161*, 246-262, doi:10.1016/j.freeradbiomed.2020.09.019.
174. Bai, Y.; Treins, C.; Volpi, V.G.; Scapin, C.; Ferri, C.; Mastrangelo, R.; Touvier, T.; Florio, F.; Bianchi, F.; Del Carro, U.; et al. Treatment with IFB-088 Improves Neuropathy in CMT1A and CMT1B Mice. *Molecular neurobiology* **2022**, *59*, 4159-4178, doi:10.1007/s12035-022-02838-y.
175. Klein, D.; Patzkó, Á.; Schreiber, D.; van Hauwermeiren, A.; Baier, M.; Groh, J.; West, B.L.; Martini, R. Targeting the colony stimulating factor 1 receptor alleviates two forms of Charcot-Marie-Tooth disease in mice. *Brain : a journal of neurology* **2015**, *138*, 3193-3205, doi:10.1093/brain/awv240.
176. Ha, N.; Choi, Y.I.; Jung, N.; Song, J.Y.; Bae, D.K.; Kim, M.C.; Lee, Y.J.; Song, H.; Kwak, G.; Jeong, S.; et al. A novel histone deacetylase 6 inhibitor improves myelination of Schwann cells in a model of Charcot-Marie-Tooth disease type 1A. *British journal of pharmacology* **2020**, *177*, 5096-5113, doi:10.1111/bph.15231.
177. Prior, R.; Verschoren, S.; Vints, K.; Jaspers, T.; Rossaert, E.; Klingl, Y.E.; Silva, A.; Hersmus, N.; Van Damme, P.; Van Den Bosch, L. HDAC3 Inhibition Stimulates Myelination in a CMT1A Mouse Model. *Molecular neurobiology* **2022**, *59*, 3414-3430, doi:10.1007/s12035-022-02782-x.
178. Patzkó, A.; Bai, Y.; Saporta, M.A.; Katona, I.; Wu, X.; Vizzuso, D.; Feltri, M.L.; Wang, S.; Dillon, L.M.; Kamholz, J.; et al. Curcumin derivatives promote Schwann cell differentiation and improve neuropathy in R98C CMT1B mice. *Brain : a journal of neurology* **2012**, *135*, 3551-3566, doi:10.1093/brain/aws299.
179. D'Antonio, M.; Musner, N.; Scapin, C.; Ungaro, D.; Del Carro, U.; Ron, D.; Feltri, M.L.; Wrabetz, L. Resetting translational homeostasis restores myelination in Charcot-Marie-Tooth disease type 1B mice. *The Journal of experimental medicine* **2013**, *210*, 821-838, doi:10.1084/jem.20122005.
180. VerPlank, J.J.S.; Gawron, J.; Silvestri, N.J.; Feltri, M.L.; Wrabetz, L.; Goldberg, A.L. Raising cGMP restores proteasome function and myelination in mice with a proteotoxic neuropathy. *Brain : a journal of neurology* **2022**, *145*, 168-178, doi:10.1093/brain/awab249.
181. Ostertag, C.; Klein, D.; Martini, R. Presymptomatic macrophage targeting has a long-lasting therapeutic effect on treatment termination in a mouse model of Charcot-Marie-Tooth 1. *Experimental neurology* **2022**, *357*, 114195, doi:10.1016/j.expneurol.2022.114195.

182. Scapin, C.; Ferri, C.; Pettinato, E.; Zambroni, D.; Bianchi, F.; Del Carro, U.; Belin, S.; Caruso, D.; Mitro, N.; Pellegatta, M.; et al. Enhanced axonal neuregulin-1 type-III signaling ameliorates neurophysiology and hypomyelination in a Charcot-Marie-Tooth type 1B mouse model. *Human molecular genetics* **2019**, *28*, 992-1006, doi:10.1093/hmg/ddy411.
183. Nicks, J.; Lee, S.; Harris, A.; Falk, D.J.; Todd, A.G.; Arredondo, K.; Dunn, W.A., Jr.; Notterpek, L. Rapamycin improves peripheral nerve myelination while it fails to benefit neuromuscular performance in neuropathic mice. *Neurobiology of disease* **2014**, *70*, 224-236, doi:10.1016/j.nbd.2014.06.023.
184. Okamoto, Y.; Pehlivan, D.; Wiszniewski, W.; Beck, C.R.; Snipes, G.J.; Lupski, J.R.; Khajavi, M. Curcumin facilitates a transitory cellular stress response in Trembler-J mice. *Human molecular genetics* **2013**, *22*, 4698-4705, doi:10.1093/hmg/ddt318.
185. Khajavi, M.; Shiga, K.; Wiszniewski, W.; He, F.; Shaw, C.A.; Yan, J.; Wensel, T.G.; Snipes, G.J.; Lupski, J.R. Oral curcumin mitigates the clinical and neuropathologic phenotype of the Trembler-J mouse: a potential therapy for inherited neuropathy. *American journal of human genetics* **2007**, *81*, 438-453, doi:10.1086/519926.
186. Franco, A.; Dang, X.; Walton, E.K.; Ho, J.N.; Zablocka, B.; Ly, C.; Miller, T.M.; Baloh, R.H.; Shy, M.E.; Yoo, A.S.; et al. Burst mitofusin activation reverses neuromuscular dysfunction in murine CMT2A. *eLife* **2020**, *9*, doi:10.7554/eLife.61119.
187. Detmer, S.A.; Chan, D.C. Complementation between mouse Mfn1 and Mfn2 protects mitochondrial fusion defects caused by CMT2A disease mutations. *The Journal of cell biology* **2007**, *176*, 405-414, doi:10.1083/jcb.200611080.
188. Sato-Yamada, Y.; Strickland, A.; Sasaki, Y.; Bloom, J.; DiAntonio, A.; Milbrandt, J. A SARM1-mitochondrial feedback loop drives neuropathogenesis in a Charcot-Marie-Tooth disease type 2A rat model. *The Journal of clinical investigation* **2022**, *132*, doi:10.1172/jci161566.
189. Rocha, A.G.; Franco, A.; Krezel, A.M.; Rumsey, J.M.; Alberti, J.M.; Knight, W.C.; Biris, N.; Zacharioudakis, E.; Janetka, J.W.; Baloh, R.H.; et al. MFN2 agonists reverse mitochondrial defects in preclinical models of Charcot-Marie-Tooth disease type 2A. *Science (New York, N.Y.)* **2018**, *360*, 336-341, doi:10.1126/science.aao1785.
190. Mo, Z.; Zhao, X.; Liu, H.; Hu, Q.; Chen, X.Q.; Pham, J.; Wei, N.; Liu, Z.; Zhou, J.; Burgess, R.W.; et al. Aberrant GlyRS-HDAC6 interaction linked to axonal transport deficits in Charcot-Marie-Tooth neuropathy. *Nature communications* **2018**, *9*, 1007, doi:10.1038/s41467-018-03461-z.
191. Benoy, V.; Van Helleputte, L.; Prior, R.; d'Ydewalle, C.; Haeck, W.; Geens, N.; Scheveneels, W.; Schevenels, B.; Cader, M.Z.; Talbot, K.; et al. HDAC6 is a therapeutic target in mutant GARS-induced Charcot-Marie-Tooth disease. *Brain : a journal of neurology* **2018**, *141*, 673-687, doi:10.1093/brain/awx375.
192. Maciel, R.; Correa, R.; Bosso Taniguchi, J.; Prufer Araujo, I.; Saporta, M.A. Human Tridimensional Neuronal Cultures for Phenotypic Drug Screening in Inherited Peripheral Neuropathies. *Clinical pharmacology and therapeutics* **2020**, *107*, 1231-1239, doi:10.1002/cpt.1718.
193. Kim, J.Y.; Woo, S.Y.; Hong, Y.B.; Choi, H.; Kim, J.; Choi, H.; Mook-Jung, I.; Ha, N.; Kyung, J.; Koo, S.K.; et al. HDAC6 Inhibitors Rescued the Defective Axonal Mitochondrial Movement in Motor Neurons Derived from the Induced Pluripotent Stem Cells of Peripheral Neuropathy Patients with HSPB1 Mutation. *Stem cells international* **2016**, *2016*, 9475981, doi:10.1155/2016/9475981.
194. Benoy, V.; Vanden Berghe, P.; Jarpe, M.; Van Damme, P.; Robberecht, W.; Van Den Bosch, L. Development of Improved HDAC6 Inhibitors as Pharmacological Therapy for Axonal Charcot-Marie-Tooth Disease. *Neurotherapeutics : the journal of the American Society for Experimental NeuroTherapeutics* **2017**, *14*, 417-428, doi:10.1007/s13311-016-0501-z.

195. Sawade, L.; Grandi, F.; Mignanelli, M.; Patiño-López, G.; Klinkert, K.; Langa-Vives, F.; Di Guardo, R.; Echard, A.; Bolino, A.; Haucke, V. Rab35-regulated lipid turnover by myotubularins represses mTORC1 activity and controls myelin growth. *Nature communications* **2020**, *11*, 2835, doi:10.1038/s41467-020-16696-6.
196. Bolino, A.; Piguet, F.; Alberizzi, V.; Pellegatta, M.; Rivellini, C.; Guerrero-Valero, M.; Noseda, R.; Brombin, C.; Nonis, A.; D'Adamo, P.; et al. Niacin-mediated Tace activation ameliorates CMT neuropathies with focal hypermyelination. *EMBO molecular medicine* **2016**, *8*, 1438-1454, doi:10.15252/emmm.201606349.
197. Guerrero-Valero, M.; Grandi, F.; Cipriani, S.; Alberizzi, V.; Di Guardo, R.; Chicanne, G.; Sawade, L.; Bianchi, F.; Del Carro, U.; De Curtis, I.; et al. Dysregulation of myelin synthesis and actomyosin function underlies aberrant myelin in CMT4B1 neuropathy. *Proceedings of the National Academy of Sciences of the United States of America* **2021**, *118*, doi:10.1073/pnas.2009469118.
198. Sahenk, Z.; Galloway, G.; Clark, K.R.; Malik, V.; Rodino-Klapac, L.R.; Kaspar, B.K.; Chen, L.; Braganza, C.; Montgomery, C.; Mendell, J.R. AAV1.NT-3 gene therapy for charcot-marie-tooth neuropathy. *Molecular therapy : the journal of the American Society of Gene Therapy* **2014**, *22*, 511-521, doi:10.1038/mt.2013.250.
199. Sahenk, Z.; Nagaraja, H.N.; McCracken, B.S.; King, W.M.; Freimer, M.L.; Cedarbaum, J.M.; Mendell, J.R. NT-3 promotes nerve regeneration and sensory improvement in CMT1A mouse models and in patients. *Neurology* **2005**, *65*, 681-689, doi:10.1212/01.wnl.0000171978.70849.c5.
200. Gautier, B.; Hajjar, H.; Soares, S.; Berthelot, J.; Deck, M.; Abbou, S.; Campbell, G.; Ceprian, M.; Gonzalez, S.; Fovet, C.M.; et al. AAV2/9-mediated silencing of PMP22 prevents the development of pathological features in a rat model of Charcot-Marie-Tooth disease 1 A. *Nat Commun* **2021**, *12*, 2356, doi:10.1038/s41467-021-22593-3.
201. Stavrou, M.; Kagiava, A.; Choudury, S.G.; Jennings, M.J.; Wallace, L.M.; Fowler, A.M.; Heslegrave, A.; Richter, J.; Tryfonos, C.; Christodoulou, C.; et al. A translatable RNAi-driven gene therapy silences PMP22/Pmp22 genes and improves neuropathy in CMT1A mice. *The Journal of clinical investigation* **2022**, *132*, doi:10.1172/jci159814.
202. Zhao, H.T.; Damle, S.; Ikeda-Lee, K.; Kuntz, S.; Li, J.; Mohan, A.; Kim, A.; Hung, G.; Scheideler, M.A.; Scherer, S.S.; et al. PMP22 antisense oligonucleotides reverse Charcot-Marie-Tooth disease type 1A features in rodent models. *The Journal of clinical investigation* **2018**, *128*, 359-368, doi:10.1172/jci96499.
203. Boutary, S.; Caillaud, M.; El Madani, M.; Vallat, J.M.; Loisel-Duwattez, J.; Rouyer, A.; Richard, L.; Gracia, C.; Urbinati, G.; Desmaële, D.; et al. Squalenoyl siRNA PMP22 nanoparticles are effective in treating mouse models of Charcot-Marie-Tooth disease type 1 A. *Communications biology* **2021**, *4*, 317, doi:10.1038/s42003-021-01839-2.
204. Lee, J.S.; Chang, E.H.; Koo, O.J.; Jwa, D.H.; Mo, W.M.; Kwak, G.; Moon, H.W.; Park, H.T.; Hong, Y.B.; Choi, B.O. Pmp22 mutant allele-specific siRNA alleviates demyelinating neuropathic phenotype in vivo. *Neurobiology of disease* **2017**, *100*, 99-107, doi:10.1016/j.nbd.2017.01.006.
205. Lee, J.S.; Lee, J.Y.; Song, D.W.; Bae, H.S.; Doo, H.M.; Yu, H.S.; Lee, K.J.; Kim, H.K.; Hwang, H.; Kwak, G.; et al. Targeted PMP22 TATA-box editing by CRISPR/Cas9 reduces demyelinating neuropathy of Charcot-Marie-Tooth disease type 1A in mice. *Nucleic acids research* **2020**, *48*, 130-140, doi:10.1093/nar/gkz1070.
206. Lee, J.S.; Kwak, G.; Kim, H.J.; Park, H.T.; Choi, B.O.; Hong, Y.B. miR-381 Attenuates Peripheral Neuropathic Phenotype Caused by Overexpression of PMP22. *Experimental neurobiology* **2019**, *28*, 279-288, doi:10.5607/en.2019.28.2.279.

207. Serfecz, J.; Bazick, H.; Al Salihi, M.O.; Turner, P.; Fields, C.; Cruz, P.; Renne, R.; Notterpek, L. Downregulation of the human peripheral myelin protein 22 gene by miR-29a in cellular models of Charcot-Marie-Tooth disease. *Gene therapy* **2019**, *26*, 455-464, doi:10.1038/s41434-019-0098-z.
208. Hai, M.; Bidichandani, S.I.; Hogan, M.E.; Patel, P.I. Competitive binding of triplex-forming oligonucleotides in the two alternate promoters of the PMP22 gene. *Antisense & nucleic acid drug development* **2001**, *11*, 233-246, doi:10.1089/108729001317022232.
209. Geisler, S.; Huang, S.X.; Strickland, A.; Doan, R.A.; Summers, D.W.; Mao, X.; Park, J.; DiAntonio, A.; Milbrandt, J. Gene therapy targeting SARM1 blocks pathological axon degeneration in mice. *The Journal of experimental medicine* **2019**, *216*, 294-303, doi:10.1084/jem.20181040.
210. Zhou, Y.; Carmona, S.; Muhammad, A.; Bell, S.; Landeros, J.; Vazquez, M.; Ho, R.; Franco, A.; Lu, B.; Dorn, G.W., 2nd; et al. Restoring mitofusin balance prevents axonal degeneration in a Charcot-Marie-Tooth type 2A model. *The Journal of clinical investigation* **2019**, *129*, 1756-1771, doi:10.1172/jci124194.
211. Morelli, K.H.; Griffin, L.B.; Pyne, N.K.; Wallace, L.M.; Fowler, A.M.; Oprescu, S.N.; Takase, R.; Wei, N.; Meyer-Schuman, R.; Mellacheruvu, D.; et al. Allele-specific RNA interference prevents neuropathy in Charcot-Marie-Tooth disease type 2D mouse models. *The Journal of clinical investigation* **2019**, *129*, 5568-5583, doi:10.1172/jci130600.
212. Ozes, B.; Moss, K.; Myers, M.; Ridgley, A.; Chen, L.; Murrey, D.; Sahenk, Z. AAV1.NT-3 gene therapy in a CMT2D model: phenotypic improvements in Gars(P278KY/+) mice. *Brain communications* **2021**, *3*, fcab252, doi:10.1093/braincomms/fcab252.
213. Nizzardo, M.; Simone, C.; Rizzo, F.; Salani, S.; Dametti, S.; Rinchetti, P.; Del Bo, R.; Foust, K.; Kaspar, B.K.; Bresolin, N.; et al. Gene therapy rescues disease phenotype in a spinal muscular atrophy with respiratory distress type 1 (SMARD1) mouse model. *Science advances* **2015**, *1*, e1500078, doi:10.1126/sciadv.1500078.
214. Shababi, M.; Feng, Z.; Villalon, E.; Sibigtroth, C.M.; Osman, E.Y.; Miller, M.R.; Williams-Simon, P.A.; Lombardi, A.; Sass, T.H.; Atkinson, A.K.; et al. Rescue of a Mouse Model of Spinal Muscular Atrophy With Respiratory Distress Type 1 by AAV9-IGHMBP2 Is Dose Dependent. *Molecular therapy : the journal of the American Society of Gene Therapy* **2016**, *24*, 855-866, doi:10.1038/mt.2016.33.
215. Schiza, N.; Georgiou, E.; Kagiava, A.; Médard, J.J.; Richter, J.; Tryfonos, C.; Sargiannidou, I.; Heslegrave, A.J.; Rossor, A.M.; Zetterberg, H.; et al. Gene replacement therapy in a model of Charcot-Marie-Tooth 4C neuropathy. *Brain : a journal of neurology* **2019**, *142*, 1227-1241, doi:10.1093/brain/awz064.
216. Presa, M.; Bailey, R.M.; Davis, C.; Murphy, T.; Cook, J.; Walls, R.; Wilpan, H.; Bogdanik, L.; Lenk, G.M.; Burgess, R.W.; et al. AAV9-mediated FIG4 delivery prolongs life span in Charcot-Marie-Tooth disease type 4J mouse model. *The Journal of clinical investigation* **2021**, *131*, doi:10.1172/jci137159.
217. Sargiannidou, I.; Kagiava, A.; Bashiardes, S.; Richter, J.; Christodoulou, C.; Scherer, S.S.; Kleopa, K.A. Intraneural GJB1 gene delivery improves nerve pathology in a model of X-linked Charcot-Marie-Tooth disease. *Annals of neurology* **2015**, *78*, 303-316, doi:10.1002/ana.24441.
218. Kagiava, A.; Karaiskos, C.; Richter, J.; Tryfonos, C.; Jennings, M.J.; Heslegrave, A.J.; Sargiannidou, I.; Stavrou, M.; Zetterberg, H.; Reilly, M.M.; et al. AAV9-mediated Schwann cell-targeted gene therapy rescues a model of demyelinating neuropathy. *Gene therapy* **2021**, *28*, 659-675, doi:10.1038/s41434-021-00250-0.
219. Ozes, B.; Myers, M.; Moss, K.; McKinney, J.; Ridgley, A.; Chen, L.; Bai, S.; Abrams, C.K.; Freidin, M.M.; Mendell, J.R.; et al. AAV1.NT-3 gene therapy for X-linked Charcot-Marie-Tooth neuropathy type 1. *Gene therapy* **2022**, *29*, 127-137, doi:10.1038/s41434-021-00231-3.
